# The role of pyroptosis in inflammatory diseases

**DOI:** 10.3389/fcell.2023.1173235

**Published:** 2023-05-12

**Authors:** Rong Chai, Ying Li, Linna Shui, Longxing Ni, Ansheng Zhang

**Affiliations:** Department of Stomatology, Xi’an International Medical Center Hospital Affiliated to Northwest University, Xi’an, Shaanxi, China

**Keywords:** pyroptosis, inflammasome, programed cell death, inflammation, dental pulp fibroblasts

## Abstract

Programmed cell death has crucial roles in the physiological maturation of an organism, the maintenance of metabolism, and disease progression. Pyroptosis, a form of programmed cell death which has recently received much attention, is closely related to inflammation and occurs via canonical, non-canonical, caspase-3-dependent, and unclassified pathways. The pore-forming gasdermin proteins mediate pyroptosis by promoting cell lysis, contributing to the outflow of large amounts of inflammatory cytokines and cellular contents. Although the inflammatory response is critical for the body’s defense against pathogens, uncontrolled inflammation can cause tissue damage and is a vital factor in the occurrence and progression of various diseases. In this review, we briefly summarize the major signaling pathways of pyroptosis and discuss current research on the pathological function of pyroptosis in autoinflammatory diseases and sterile inflammatory diseases.

## 1 Introduction

**FIGURE 1:**
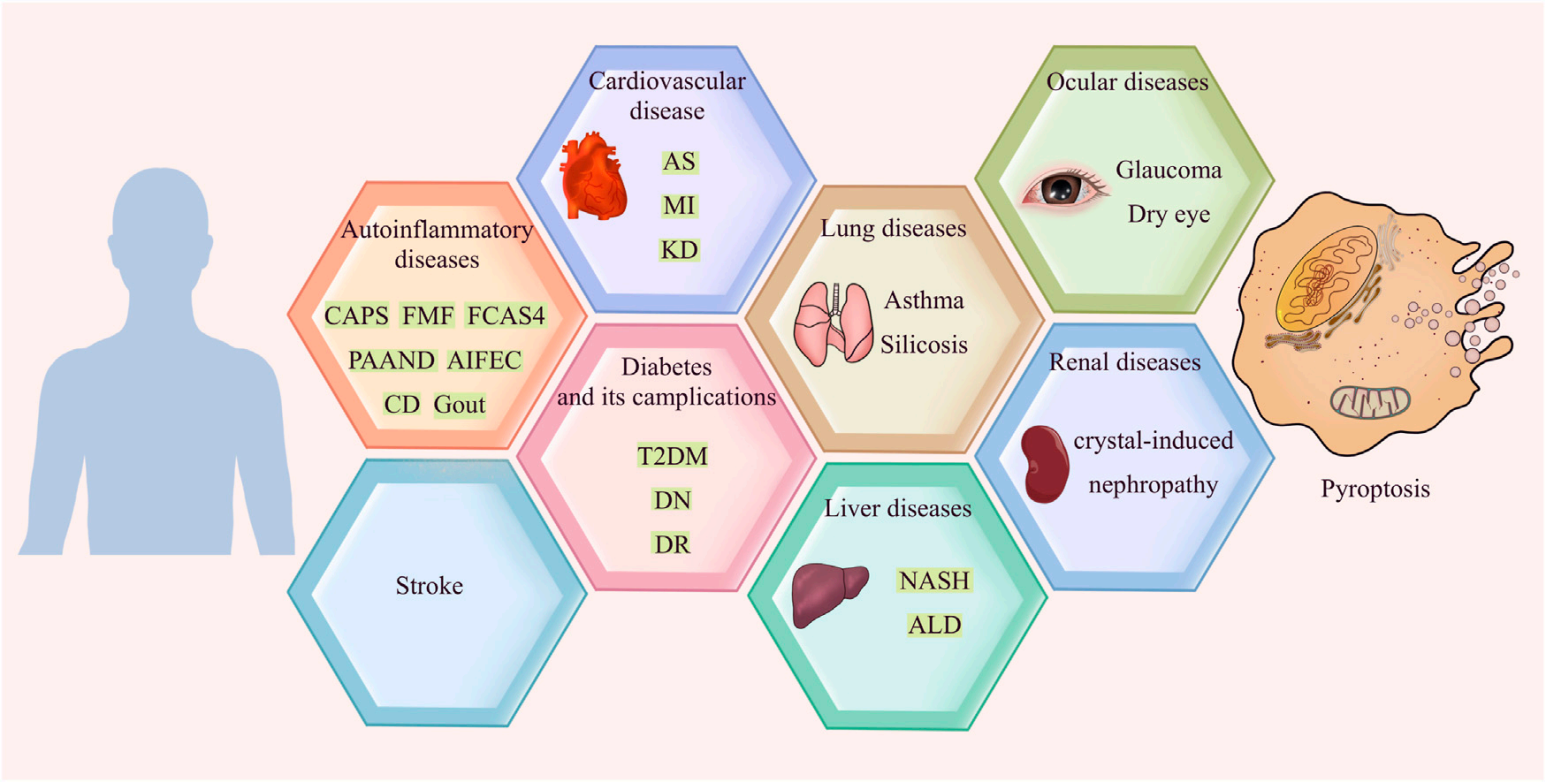
Pyroptosis in inflammatory diseases.

Apoptosis was once considered the only form of programmed cell death. However, with the deepening of research, more types of molecularly controlled cell death have been identified; pyroptosis is one of them. In 1992, a study observed that *Shigella* flexneri induces the lytic death of host macrophages, which was then identified as apoptosis (Zychlinsky et al., 1992). It was not until the 21st century that an important work demonstrated that this form of death was caspase-1 dependent, and identified this phenomenon as a new form of cell death for the first time (Cookson and Brennan, 2001). The morphology of pyroptosis is characterized by cell membrane lysis and massive production of inflammatory factors (Zhang et al., 2018a). During pyroptosis, interleukin-1β (IL-1β) precursor and interleukin-18 (IL-18) precursor are cleaved into IL-1β and IL-18, which are then released in large quantities. The secreted IL-1β then recruits and activates other cells in surrounding tissues, thereby causing the production of chemokines, inflammatory factors and adhesion molecules. The resulting cascade effect amplifies the inflammatory response, which may lead to tissue damage and other detrimental effects (Dutta et al., 2012). Inflammation is a protective response of the immune system to pathogens, damaged cells and other harmful stimuli that is driven by innate phagocytes such as macrophages and neutrophils, which produce inflammatory factors, clear out the pathogens and necrotic cells, and initiate tissue repair. However, an uncontrolled inflammatory response can cause extensive tissue damage and cell death through various pathways. In this review, we summarize the latest research on the role of pyroptosis in various inflammatory diseases and the underlying mechanisms of pyroptosis (Figure 1). Pyroptosis is still a nascent field but will undoubtedly gain considerable attention in the next few years. Further advances in understanding the relationship between pyroptosis and inflammation and associated signals will be important for selective intervention in pyroptosis for therapeutic purposes.

## 2 Mechanisms of pyroptosis

**FIGURE 2:**
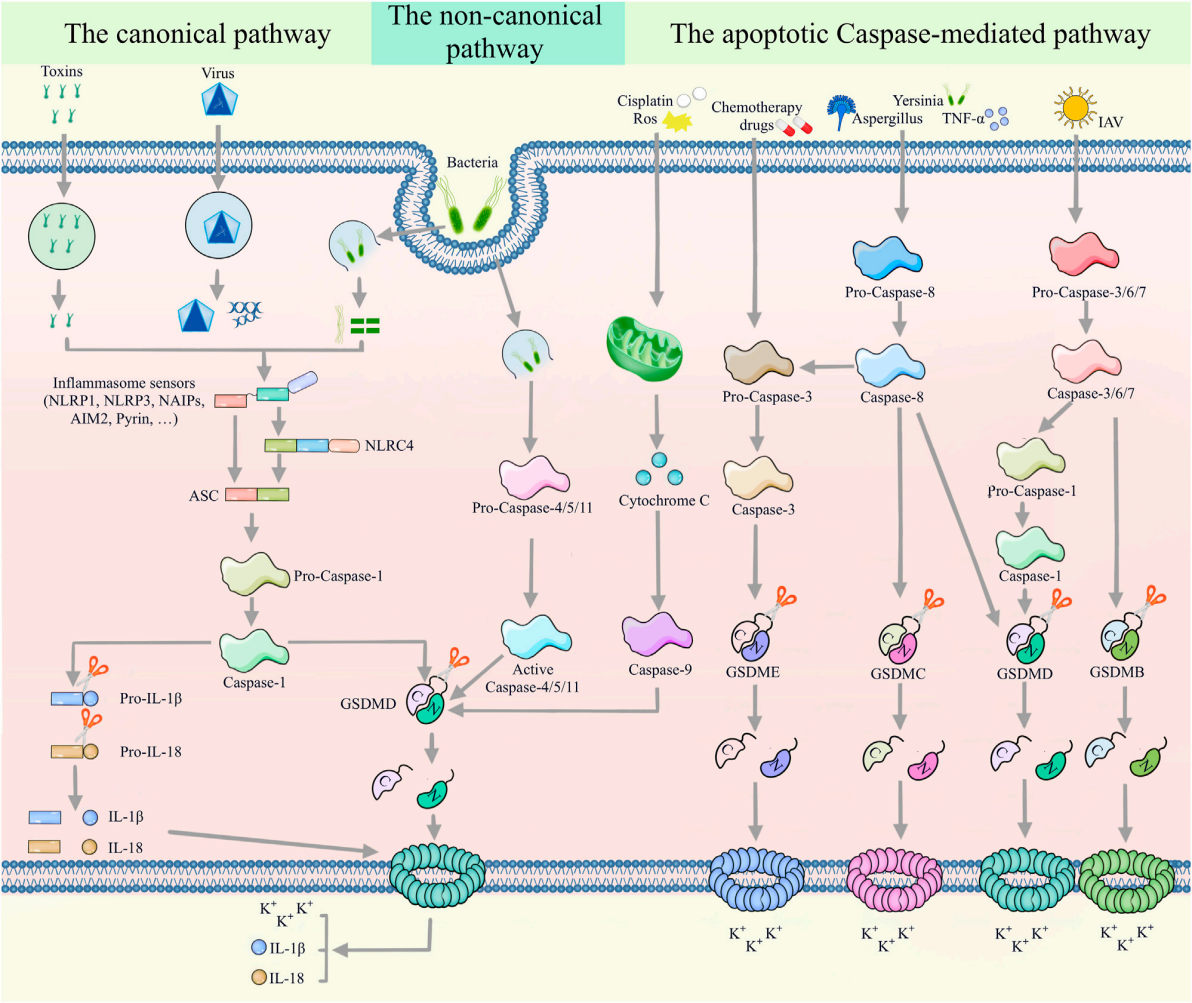
Multiple assembly mechanisms of canonical, non-canonical, and apoptotic caspases-mediated pathways. In the canonical pyroptotic pathway, inflammasome sensors (such as NLRP1, AIM2, NLRP3, NAIP and pyrin) detect diverse pathogenic signals and activate caspase-1 through the ASC or NLRC4 adaptor. Activated caspase-1 promotes the cleavage of pro-IL-1β and pro-IL-18, as well as GSDMD. Large amounts of cleaved IL-1β and IL-18 are released to the extracellular space through the membrane pores formed by the N-terminus of GSDMD. Furthermore, bacterial LPS can promote the cleavage of GSDMD by activating caspase-4/5/11 in a non-classical pyroptotic pathway. In the apoptotic caspases-mediated pathway, caspase-9/GSDMD, caspase-3/GSDME, caspase-8/GSDMC, caspase-8/caspase-3/GSDME, caspase-8/GSDMD, caspase-3/6/7/caspase-1/GSDMD and caspase-3/6/7/GSDMB signaling pathways mediate pyroptosis.

**FIGURE 3:**
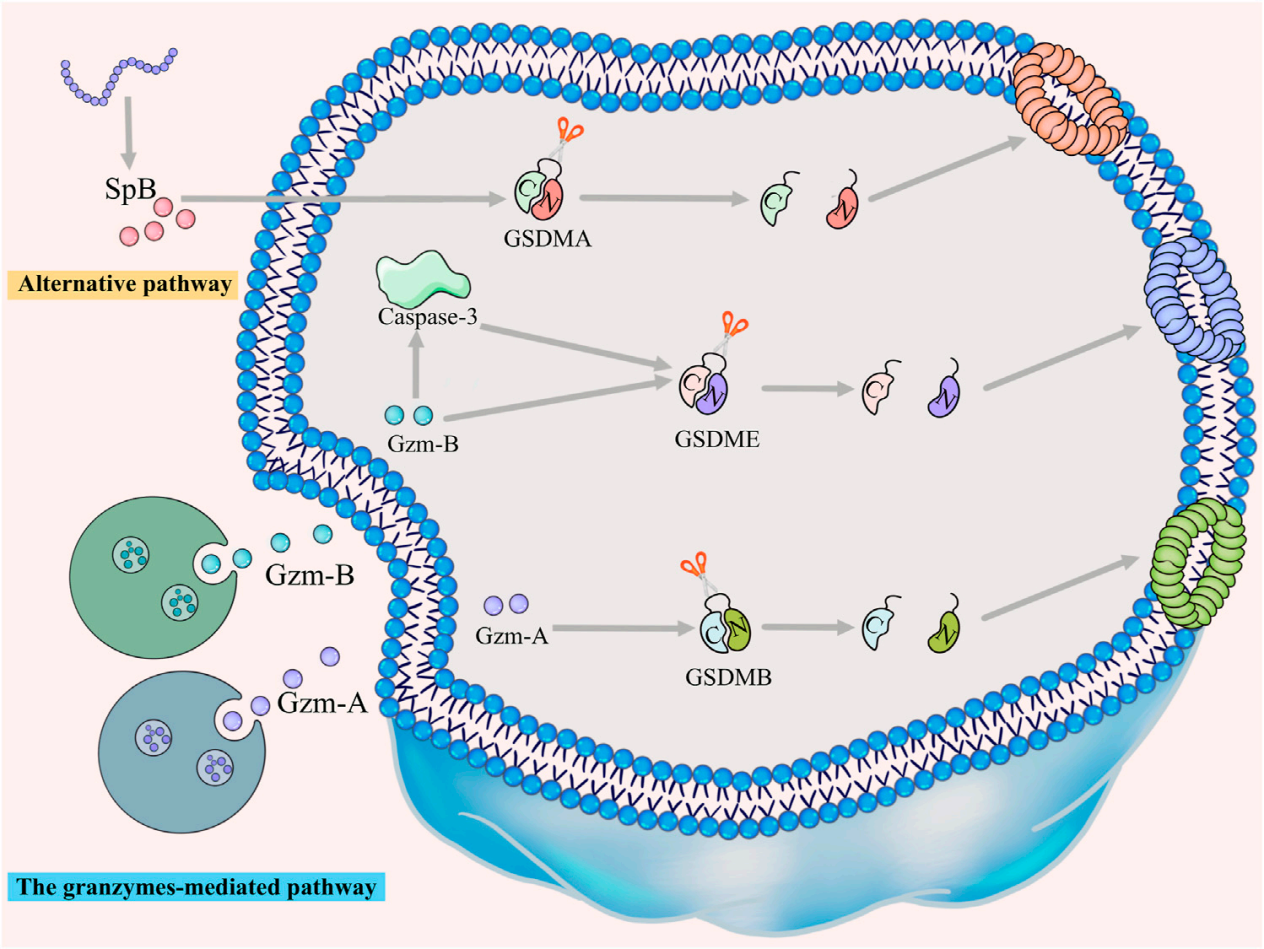
The granzymes-mediated and alternative pathways. GZMA/GSDMB, GZMB/GSDME and GZMB/caspase-3/GSDME signaling pathways trigger granzymes-mediated pyroptosis. Furthermore, GSDMA can be directly cleaved by SpB to trigger pyroptosis, a pathway that has not yet been classified.

For many years, the consensus was that pyroptosis is mediated by the canonical and non-canonical pathways. However, recent studies have unearthed apoptotic caspases-mediated pathway, granzymes-based pathway and several other pyroptotic pathways that have not been categorized until now (Figures 2, 3). Gasdermin proteins are the most important executioners of pyroptosis, which can be cleaved by upstream caspases or granzymes or directly by the pathogen, then triggering pyroptosis. There are six GSDM protein family members, and five of them are closely related to pyroptosis, namely, GSDMA-GSDME (Wang et al., 2017). Earlier studies suggested that GSDMD and GSDME were the final executors of pyroptosis, but with the deepening of research, the role of GSDMA, GSDMB and GSDMC in pyroptosis was gradually mentioned. Caspases can be categorized into inflammatory and apoptotic caspases by their function. Inflammatory caspases include caspases-1/4/5/11, which are mainly involved in the canonical pathway and non-canonical pathway, and promotes protective immunity. Apoptosis caspases contains caspases-2/3/6/7/8/9/10, which can not only initiate apoptosis, but also initiate pyroptosis (Thornberry et al., 1997; Shi et al., 2015). In the following, we will discuss the various signaling pathways of pyroptosis in more detail.

### 2.1 The canonical pathway

The canonical pathway is the most important way of pyroptosis. When the body is stimulated or infected by pathogenic bacterias, it rapidly responds by assembling the inflammasome complex, activates caspase-1, and triggers pyroptosis. The inflammasome is a protein complex composed of pattern recognition receptors (PRRs), inflammatory cysteine proteases and apoptosis-associated speck-like protein containing a CARD (ASC) (Platnich and Muruve, 2019). There are four main types of PRRS, nucleotide-binding oligomerization domainlike receptor (NLR), C-type lectin receptors (CLR), absent in melaroma 2 like receptor (ALR) and toll-like receptor (TLR) assemble together with procaspase-1 precursor to form inflammasome. There are five main types of inflammasomes in the canonical pathway:NLRP3 inflammasome, AIM2 inflammasome, NLRP1 inflammasome, PYRIN inflammasome, and NLRC4 inflammasome (Martinon et al., 2002; Rathinam et al., 2012). These inflammsomes recruit and activate pro-caspase-1 in response to viral and bacterial motifs or other pathogenic activities (Wang et al., 2019a; Mitchell et al., 2019; Xia et al., 2019). Activated caspase-1 then cleaves pro-IL-1β and pro-IL-18 into the active IL-1β and IL-18 (Fang et al., 2020). In addition, activated caspase-1 can also disassemble GSDMD protein into a hydrophobic N-terminal domain and a hydrophilic C-terminal domain. The hydrophobic N-terminal domain binds to and accumulates on the inner side of the cell membrane, and forms numerous pores that allow the outflow of IL-18 and IL-1β, which trigger the inflammation (Ding et al., 2016; Evavold et al., 2018; Zhang et al., 2021a). Recent studies have shown that not only GSDMD but also GSDMB can be cleaved by caspase-1 to trigger pyroptosis (Panganiban et al., 2018). However, Sara S Oltra et al. hold a different view, and their study proves that the N-terminal fragments of GSDMB isoforms which can be activated and cleaved by caspases does not have the ability to form pores in the cell membrane and thus fail to trigger pyroptosis (Oltra et al., 2023).

### 2.2 The non-canonical pathway

The non-canonical pyroptosis pathway is initiated by caspase-4/5/11 that can directly recognize bacterial LPS and undergo oligomerization, thereby cleaving GSDMD and triggering pyroptosis (Wandel et al., 2020). Like the canonical pathway, activated caspase-11 also cleaves GSDMD to generate pores in the cell membrane. Different from the canonical pathway, activated caspase4/5/11 can not only complete the assembly of the inflammasome complex in the presence of ASC, activate caspase1, and induce the classical pathway, thereby further expanding the inflammatory response, but also activate Pannexin-1 (Panx-1) and cause ATP release and potassium efflux (Yang et al., 2015; Aglietti and Dueber, 2017). In addition, recent works have suggested that the binding of GSDMB to the CARD domain of caspase-4 results in the oligomerization of caspase-4 protein, which promotes GSDMD cleavage and then triggers non-canonical pyroptosis (Chen et al., 2019).

### 2.3 The apoptotic caspases-mediated pathway

Recent studies have shown that apoptosis caspases can also activate pyroptosis. Caspase-3 generates pores in the cell membrane by cleaving the GSDME protein (Wang et al., 2017). The expression level of GSDME determines whether the cell changes from apoptosis to pyroptosis. In the cells with high expression of GSDME, the use of chemotherapy drugs that can activate caspase-3 to induce pyroptosis, while in the cells with low expression of GSDME, secondary necrosis occurs after apoptosis (Rogers et al., 2017; Wang et al., 2017). With the increasing focus on apoptotic caspases, caspase-8, another apoptotic caspases, was shown to initiate pyroptosis by activating GSDMD cleavage during *Yersinia* or Aspergillus fumigatus infection (Orning et al., 2018; Sarhan et al., 2018; Wang et al., 2022a). Moreover, caspase-8 can also activate and cleave GSDMC at different sites depending on different stimuli, thereby transforming TNF-α-induced apoptosis into pyroptosis (Hou et al., 2020; Zhang et al., 2021b). In addition, caspase-8 can also be activated by TNF-α, which in turn initiates caspase-3/GSDME-mediated pyroptosis (Wu et al., 2023). A recent study demonstrated that caspas-6 and -7 could induce pyroptosis by cleaving GSDMB(Chao et al., 2017). During influenza A virus infection, caspase-6 ccould trigger pyroptosis through the ZBP-1-NLRP3-caspase-1-GSDMD signaling pathway (Zheng et al., 2020). In addition, caspase-9 could not only be activated by cytoplasm cyt-c to cleave GSDMD to trigger pyroptosis, but also through the CAPN1/CAPN2-BAK/BAX/caspase-3 signaling pathway to cleave GSDME to trigger pyroptosis (Dai et al., 2022; Li et al., 2022).

### 2.4 The granzymes-mediated pathway

Several granzymes-mediated pathways of pyroptosis have also been reported in recent years. lymphocyte-derived granzyme A (GZMA) can activate pyroptosis by cleavage of GSDMB and further kill GSDMB-positive cells (Zhou et al., 2020). Sara S Oltra et al. demonstrated that immune cell-derived GZMA can cleave all isoforms of GSDMB, but only the isoforms containing exon 6, namely, GSDMB3-4, can trigger pyroptosis (Oltra et al., 2023). Granzyme B could not only directly cleave GSDME to trigger pyroptosis, but also activate caspase-3-independent pyroptosis through the caspase-3-GSDME signaling pathway (Zhang et al., 2020).

### 2.5 Alternative pathways

GSDMA also triggers pyroptosis but has not been assigned to any of above four categories. GSDMA protein is expressed in epithelial cells of skin, tongue, esophagus, stomach, breast and umbilical cord (Wu et al., 2009; Tanaka et al., 2013). SpB virulence factor secreted by group A *streptococcus* (GAS) can directly cleave GSDMA after GIn246 to trigger pyroptosis in keratinocytes (Deng et al., 2022; LaRock et al., 2022). Dead keratinocytes could prevent the invasion of GAS into the deep tissues of the body (Nakagawa et al., 2004).

## 3 The role of pyroptosis in autoinflammatory diseases

Systemic autoinflammatory disorders (SAIDs) are mediated by a dysfunctional innate immune system, and they share certain core phenotypic manifestations, including recurrent fever, cutaneous perturbations, chest or abdominal pain, lymphadenopathy, vasculopathy and musculoskeletal symptoms (McDermott et al., 1999). The mechanistic basis of SAIDs is the recognition of pathogen-associated molecular patterns (PAMPs) by innate immune cells via PRRs, and the activation of downstream signaling pathways in conjunction with damage-associated molecular patterns (DAMPs) (Ben-Chetrit et al., 2018). Pyroptosis is a vital component of the immune system, and inflammasomes, such as NLRP3, pyrin and NLRC4, take a leading role in the activation of the canonical pyroptosis pathway. In addition, aberrant activation of inflammasomes is also a key driver of SAIDs. Pyroptosis may also have a causative role in SAIDs. Data supports the idea that a lack of GSDMD does not affect pro-IL-1β activation into IL-1β but rather prevents the outflow of mature IL-1β, which indicates that the systemic inflammatory response in SAID patients is caused by excessive cytokine release from the pores during pyroptosis (Kayagaki et al., 2015; Shi et al., 2015). However, since inflammatory factors can also be produced by pathways other than pyroptosis (Mitchell et al., 2019), it cannot be ascertained whether the reduced inflammatory response following GSDMD knockout is due to the inhibition of pyroptosis or merely to reduced IL-1β release (Bulek et al., 2020). Consequently, the mechanistic function of pyroptosis in SAIDs should be further explored.

### 3.1 The NLRP3-related SAIDs

SAIDs related to the NLRP3 inflammasome include cryopyrin-associated periodic syndrome (CAPS), gout and Crohn’s disease. CAPS is a series of autosomal dominant systemic autoinflammatory diseases resulting from mutations in the NLRP3 gene. The three clinical subtypes of CAPS are NOMID (also known as chronic infantile neurocutaneous and joint syndrome), Muckle–Wells syndrome (MWS), and familial cold autoinflammatory syndrome (FCAS). These subtypes share common indications such as fever, skin and joint-related symptoms. Brydges et al. discovered a caspase-1-related form of cell death in a mouse model of FCAS, most likely pyroptosis, which induced inflammation (Brydges et al., 2013). Furthermore, Claudia et al. reported that LPS can induce pyroptosis in the monocytes of patients with CAPS, thereby aggravating the inflammatory response (Semino et al., 2018). In addition, wedelolactone promotes phosphorylation at the Ser/Thr site of NLRP3 and inhibits inflammasome activation and pyroptosis by activating protein kinase A (PKA) signaling (Baroja-Mazo et al., 2014). Mutations affecting this NLRP3 site can lead to its constitutive activation, thereby triggering pyroptosis and subsequently the inflammatory symptoms of NOMID. Knocking out GSDMD in a mouse model of NOMID obviously reduced the symptoms of leukocytosis and anemia, indicating that GSDMD is a novel therapeutic target for NOMID (Xiao et al., 2018).

### 3.2 The pyrin-related SAIDs

The pyrin-associated SAIDs include familial mediterranean fever (FMF), pyrin-associated auto-inflammation with neutrophilic dermatosis (PAAND) and livedoid ulcerative dermatitis (Papin et al., 2000; Moghaddas et al., 2017; Demircan et al., 2020), which are mainly caused by MEFV gene mutations that result in excessive activation of pyrin. Furthermore, the pathogenesis of FMF and PAAND is bound up with pyroptosis. FMF is a group of recessive genetic diseases that manifest as chronic fever, rash, serositis, arthritis, etc. In 2015, two independent groups demonstrated that caspase-1 p10/p20 tetramers recruited and activated by pyrin can process pro-IL-1β and pro-IL-18 into their activated forms (IL-1β and IL-18). The massive increase in the amount of IL-1β and IL-18 due to caspase-1-induced pyroptosis drives the inflammatory symptoms in FMF (Kayagaki et al., 2015; Shi et al., 2015). Apart from the systemic inflammatory response triggered by extracellular cytokines, ASC speckles generated by pyroptosis exhibit “prion-like” characteristics and further promote inflammation (Baroja-Mazo et al., 2014; Franklin et al., 2014). The microtubule polymerization inhibitor colchicine depolymerizes microtubules and then phosphorylates pyrin through the guanine nucleotide exchange factor (GEF)-H1-RhoA-PKN1/2 signaling pathway (Magnotti et al., 2019). Therefore, colchicine has long been considered an effective treatment for FMF and gout (Goldfinger, 1972). The main clinical manifestations of PAAND are lower age of onset, recurrent neutrophilic dermatitis, periodic fever, arthralgia, myalgia or myositis, etc. Unlike FMF, the pathogenic basis of PAAND is a gain-of-function mutation at specific sites of the MEFV gene, S242R and E244K, which produces constitutively activated pyroproteins, resulting in excess IL-1β and IL-18 release and GSDMD-mediated pyroptosis. Increased levels of pyroptosis have been detected in the peripheral blood mononuclear cells of patients with PAAND (Gao et al., 2016; Masters et al., 2016; Moghaddas et al., 2017; Hong et al., 2019). Pyroptosis increases the load of inflammatory factors in the skin of PAAND patients, eventually leading to neutrophilic dermatosis and inflammation (Moghaddas et al., 2017).

### 3.3 The NLRC4-related SAIDs

The representative NLRC4-related SAID is autoinflammation with infantile enterocolitis (AIFEC), which is primarily manifested as severe and chronic autoinflammation, macrophage activation syndrome, infantile enterocolitis, and organ-specific symptoms secondary to lympho-histiocytic inflammation. Patients who survive this autoimmune disease in infancy have a short stature and exhibit anemia in adulthood (Canna et al., 2014; Romberg et al., 2014). AIFEC is caused by heterozygous gain-of-function mutations in NLRC4. There are now been demonstrated AIFEC-associated mutations: V341A, T337S, T337N and S171F (Canna et al., 2014; Romberg et al., 2014; Canna et al., 2017; Liang et al., 2017). Studies show that physical and emotional stress are the triggers for AIFEC (Lin et al., 2020a). *Salmonella* or *Pseudomonas* cells induced pyroptosis in the macrophages of AIFEC patients *in vitro* by stimulating NLRC4 (Romberg et al., 2014). In addition, there is another NLRC4-related SAID, which is familial cold autoinflammatory syndrome 4 (FCAS4). The symptoms of FCAS4 are relatively mild, mainly characterized by recurrent episodes of fever, arthralgia, and cold-induced urticarial rashes in childhood. A recent study demonstrated that FCAS4 is caused by a heterozygous p. Ser445pro NLRC4 mutation (Gil-Lianes et al., 2023). This variant was able to recruit ASC and assemble constitutively active oligomeric inflammasomes and resulted in increased IL-18 expression. Further studies are needed to reveal the relationship and mechanism between FCAS and pyroptosis.

### 3.4 The polygenic and multifactorial SAIDs

Polygenic and multifactorial SAIDs are caused by immunological, genetic and environmental factors. Common diseases include Behcet’s disease (BD), Crohn’s disease (CD), gout, chronic aseptic osteomyelitis, chronic relapsing multifocal osteomyelitis, juvenile idiopathic systemic arthritis, etc. Gout is caused by the deposition of monosodium urate (MSU) crystals or dehydrated calcium pyrophosphate (CPPD) crystals in the joints, which clinically manifests as fever and severe joint pain and swelling. One study showed that NLRP3 activation by MSU crystals resulted in marked neutrophil infiltration and the onset of pyroptosis in a rat model of gout. Bromodomain-containing protein 4 (BRD4) knockdown significantly reduced p65 NF-κB signaling and NLRP3 inflammasome activation, which reduced joint swelling and synovial inflammation. The authors therefore concluded that BRD4 is closely related to the pyroptosis cascade in MSU-induced acute gouty arthritis, and JQ-1 or siRNA-mediated knockdown can mitigate inflammation by inhibiting the BRD4/NF-κB/NLRP3/GSDMD axis (Lin et al., 2020b; Hao et al., 2020). In addition, Tian et al. detected an increase in the levels of inflammatory factors and pyroptosis markers in a mouse model of oxygenated potassium (PO) and MSU-induced gout, which were downregulated by treating the mice with disulfiram (Tian et al., 2022). Another work demonstrated that gallic acid limited the activation of NLRP3 inflammasome and Nrf2 signaling-dependent pyroptosis, and alleviated NLRP3-mediated gouty arthritis by inhibiting ROS production (Lin et al., 2020a). In addition, Baeckein E (BF-2) also suppressed the inflammatory response in a mouse model of gout by inhibiting the pyroptosis of macrophages (Lin et al., 2021). Zhao et al. hypothesized that the onset of gout is bound up with pyroptosis, and that NLRP3, in particular, acts as a key character in the progression from hyperuricemia to gout. Therefore, screening for novel pyroptosis inhibitors for treating gout should pay attention to upstream factors such as NLRP3 (Zhao et al., 2022).

The clinical manifestations of CD are abdominal pain, diarrhea and other gastrointestinal symptoms, and the pathological manifestations are abnormal intestinal mucosa and excessive inflammatory response. The morbidity of CD has expanded in recent decades (Clough et al., 2020). The GSDME N-terminus has been observed in the inflamed intestinal mucosal epithelium of CD patients but not in the normal colonic mucosa of healthy controls. In addition, Gsdme−/− mice and wild-type (WT) littermate controls were treated with 2,4,6-trinitrobenzenesulfonic acid (TNBS) to induce colitis, and the results showed that Gsdme−/− mice exhibit less severe intestinal inflammation than WT controls do (Tan and Chen, 2021b). Similarly, Bulek et al. also suggested that mice lacking the GSDMD gene were protected from dextran sulfate sodium (DSS)-induced colitis (Bulek et al., 2020). In addition, one study demonstrated that C-type lectin (Mincle)-induced macrophage pyroptosis is the basis of intestinal inflammation in CD patients, and the Mincle/Syk axis is a probable target for CD therapy (Gong et al., 2020). Another recent study showed that miR-200c can reduce inflammation levels and NLRP3-induced MODE-K cell pyroptosis *in vitro*, by targeting NIMA-related kinase 7 (NEK7), and so improve DSS-induced intestinal injury in mice (Wu et al., 2022). Taken together, the inflammasome can activate GSDMD-mediated pyroptosis through different signaling pathways, thereby promoting and amplifying intestinal inflammation. Interestingly, a recent study showed that GSDMD-mediated formation of pores in the cell membrane resulted in massive outflow of potassium ions, which further inhibited the cGAS-STING signaling pathway and mitigated intestinal inflammation (Ma et al., 2020). In conclusion, while GSDMD-dependent macrophage pyroptosis promotes and amplifies intestinal mucosal inflammation, there is evidence of an anti-inflammatory role as well.

## 4 The role of pyroptosis in sterile inflammatory diseases

### 4.1 Pyroptosis in cardiovascular diseases

Recent studies have demonstrated that pyroptosis-related factors, such as NLRP3 and caspase-1, are involved in angiogenesis, myocardial hypertrophy, plaque formation in arterial walls, endothelial damage, myocardial fibrosis and other pathophysiological processes that form the basis of multiple cardiovascular diseases, including myocardial infarction (MI)/reperfusion, atherosclerosis (AS) and Kawasaki disease (KD).

AS is a common cardiovascular disease characterized by thickening and hardening of blood vessel walls. Vascular endothelial cells, smooth muscle cells and macrophages undergo pyroptosis during different stages of AS (Cypryk et al., 2018). In addition, caspase-1 and NLRP3 have been identified as markers of AS-related inflammation (Xu et al., 2018a). Atorvastatin, a lipid-lowering drug that is prescribed for AS patients, can inhibit pyroptosis by downregulating the mRNA and protein levels of NLRP3 and GSDMD, which is likely the basis of its anti-atherosclerotic effect (Wu et al., 2020). Furthermore, dihydromyricetin, pyrogallol, β-hydroxybutyrate, antimicrobial peptide LL-37, ROS scavengers, MCC950 and other drugs can also exert an anti-atherosclerotic effect by inhibiting pyroptosis. Several non-coding RNAs that regulate pyroptosis during AS have been identified (Hu et al., 2014; Coll et al., 2015; Youm et al., 2015; Hu et al., 2018; Oh et al., 2020), including miR-22, miR-103, miR-125a-5p and miR-30c-5p (Li et al., 2018a; Wang et al., 2019b; Wang et al., 2020), of which miR-125a-5p exerts a pro-pyroptotic effect in vascular endothelial cells (Zhaolin et al., 2019). Although their regulatory role in pyroptosis is well-established, the detailed roles of these non-coding RNAs and the underlying mechanisms need further research.

MI is a lethal coronary artery disease that is responsible for more than one-third of deaths worldwide each year. During myocardial ischemia–reperfusion, vascular endothelial cells, myocardial fibroblasts and macrophages undergo pyroptosis, which releases massive amounts of inflammatory factors and metabolites that aggravate myocardial injury. Vascular endothelial injury is widely recognized as an initiating factor in cardiovascular pathologies. Inhibiting caspase-1 activation in a mouse model of hindlimb ischemia attenuated endothelial cell pyroptosis and increased their survival via VEGFR-2 signaling, which in turn improved angiogenesis and ischemic outcomes (Lopez-Pastrana et al., 2015). Likewise, another study confirmed that decreasing the level of NLRP3 activation attenuated oxidative stress and pyroptosis, thereby reducing epithelial cell dysfunction (Yang et al., 2020). Cardiac macrophages also have a crucial role in the inflammation induced by ischemia–reperfusion injury. DAMPs are produced in large quantities during reperfusion activate caspase-1 to generate pyroptosis in macrophages, and the subsequent outflow of IL-1β and IL-18 attracts large numbers of inflammatory cells to aggregate and amplify the inflammation (Xi et al., 2016). Furthermore, above-normal amounts of NLRP3 and caspase-1 have been detected in the coronary plaques of patients with acute coronary syndrome (ACS) (Lee et al., 2017). These results suggest that inhibiting macrophage pyroptosis can mitigate the inflammation, and thus alleviate the symptoms of ischemia-reperfusion injury.

The pathogenesis of Kawasaki disease, cardiac hypertrophy and diabetic cardiomyopathy is also closely related to pyroptosis. Jia et al. found that the ASC, caspase-1, IL-1β, IL-18, GSDMD and lactate dehydrogenase (LDH) were significantly elevated in the serum of KD patients in contrast to healthy controls, suggesting that pyroptosis is a key driver of coronary endothelial cell injury in KD. Furthermore, human umbilical vein endothelial cells (HUVECs) co-cultured with THP1 cells that were pre-treated with the serum of KD patients underwent pyroptosis following NLRP3 inflammasome activation (Jia et al., 2019). Cardiac hypertrophy is a clinically refractory disease with a complex molecular mechanism (Hou and Kang, 2012; Tham et al., 2015). A mouse model of cardiac hypertrophy has been established using transverse aortic constriction (TAC) that exerts pressure on the heart. In addition, cardiomyocyte hypertrophy can be simulated *in vitro* using cardiomyocyte angiotensin II (Ang II) (Heger et al., 2016). Murine cardiomyocytes treated with Ang II expressed significantly higher levels of caspase-1 and IL-1β compared to control cells (Rayamajhi et al., 2013). Co-administration of a caspase-1 inhibitor along with Ang II attenuated the thickening effect of the latter, which indicates that caspase-1-induced pyroptosis is closely related to cardiac hypertrophy and therefore a promising therapeutic target (Franchi et al., 2006). In summary, the pyroptosis of vascular endothelial cells and macrophages is an important pathological mechanism in various cardiovascular diseases.

### 4.2 Pyroptosis in diabetes and its complications

Type 2 diabetes (T2DM) is a chronic metabolic disease that clinically manifests as hyperglycemia and insufficient insulin secretion. Recent works indicate that islet inflammation is the underlying cause of progressive β-cell dysfunction and insulin resistance (IR). In addition, there is evidence that NLRP3 inflammasome-induced pyroptosis has a key role in the pathological mechanism of T2DM(Chang et al., 2021). One study showed that hyperglycemia increased mitochondrial metabolism through the advanced glycation end products (AGE) pathway, protein kinase C (PKC) and other pathways, and the high level of ROS generated during this process induced pyroptosis in the insulin-secreting β cells (Babel & Dandekar, 2021). Furthermore, mitochondrial ROS levels are significantly increased in chronic hyperglycemia patients with poor glycemic control, which results in more severe pyroptosis and inflammatory responses (Iannantuoni et al., 2019). Morikawa et al. detected massive pyroptosis-related signaling pathway proteins and macrophage aggregates in the pancreatic β cells and capillaries of diabetics (Morikawa et al., 2018). On the other hand, Lin et al. found that inhibiting the pyroptosis pathway significantly increased the expression levels of insulin sensitivity-related genes and the secretion of insulin-sensitizing hormones (Lin et al., 2020b). In addition, miR-23a-3p alleviated the symptoms of T2DM in a rat model by inhibiting pyroptosis through its target gene NEK7 (NIMA-related kinase 7), which is a crucial regulator of the NLRP3 inflammasome (Chang et al., 2021).

Diabetic nephropathy (DN) is one of the crucial causes of chronic kidney disease in China and the most common diabetic complication. Shahzad et al. observed that knocking out caspase-1 in a mouse model of diabetes inhibited inflammasome activation and attenuated symptoms of DN, thus indicating a pathological role of pyroptosis (Shahzad et al., 2016). The same group observed a significant upregulation of IL-1β in diabetic mice and found that knocking down NLRP3/caspase-1 genes in bone marrow-derived cells inhibited DN progression (Shahzad et al., 2015). Likewise, Wu et al. reported that NLRP3 gene silencing in an STZ-induced diabetic mouse model inhibited renal inflammation and fibrosis by alleviating oxidative stress (Wu et al., 2018). Furthermore, Liu et al. showed that silencing the TLR4 gene in podocytes inhibited caspase-3 activation and attenuated oxidative stress by reducing intracellular ROS levels, which in turn reduced the production of IL-1β, IL-18, TGF-β1 and TNF-α (Liu et al., 2018). Oxidative stress in the kidney acts as a key character in the progression of DN, and studies increasingly show that ROS production can further activate the NLRP3 inflammasome in renal tissues (Jha et al., 2016; Wei and Szeto, 2019). Thioredoxin-interacting protein (TXNIP) promotes oxidative stress and pyroptosis by inhibiting the antioxidant enzyme thioredoxin reductase (TRX). A previous study demonstrated that the TXNIP signaling pathway has a key role in promoting DN progression (An et al., 2020). Furthermore, Han et al. found that excessive activation of TXNIP in inflamed renal tissues initiated the NLRP3 inflammasome pathway to promote pyroptotic renal injury (Han et al., 2018). In conclusion, pyroptosis activated by the NLRP3 inflammasome is bound up with the occurrence of DN and is, therefore, a potential therapeutic target.

Diabetic retinopathy (DR) is another common complication of diabetes. Prolonged hyperglycemia induces GSDMD-induced pyroptosis, which has a crucial role in the occurrence and progression of DR. Gan et al. demonstrated that the hyperglycemic state promoted pyroptosis of human retinal progenitor (HRP) cells through the NLRP3-caspase-1-GSDMD signaling axis, which led to the deprivation of retinal pericytes (Gan et al., 2020). Yu et al. further suggested that caspase-1-mediated pyroptosis occurs in HRP cells subjected to the serum albumin-induced DR state (Yu et al., 2021).

### 4.3 Pyroptosis in lung diseases

Asthma is a chronic inflammatory disease of the respiratory system with complex etiology. Genome-wide association studies (GWAS) in recent years have shown that polymorphisms of the GSDMA and GSDMB genes are closely related to the occurrence of asthma (Moffatt et al., 2007; Yu et al., 2011; Kang et al., 2012). In fact, GSDMB is highly expressed in the airway epithelial cells of asthmatic individuals, indicating that activated GSDMB may induce caspase-mediated pyroptosis in these cells and trigger asthma. Furthermore, a splice variant of GSDMB (rs11078928) is related to a lower risk of asthma since the deletion of a key exon in the GSDMB transcript neutralizes its ability to induce pyroptosis (Panganiban et al., 2018). Another study demonstrated that caspase-4/11-induced non-canonical pyroptosis is a major cause of asthma, and that prostaglandin E2 (PGE2) exerts its anti-inflammatory effects and promotes tissue repair by targeting this pathway (Zaslona et al., 2020).

Silicosis is an interstitial lung disease caused by chronic exposure to crystalline silica dust and is characterized by accumulation of silicon nodules and diffuse pulmonary fibrosis. The inhaled silica particles are phagocytosed by alveolar macrophages (AMs), which then die and release the intracellular silica. The surrounding AMs absorb the released silica, thus forming a vicious cycle (Joshi and Knecht, 2013; Tan and Chen, 2021a). Furthermore, the dying AMs also release a large amount of inflammatory factors that trigger lung inflammation (McGrath et al., 2015). Finally, accumulation of dead AMs in the damaged lung tissue leads to extensive fibrosis (Kehlet et al., 2018; Quan et al., 2019). Song et al. showed that NLRP3 and its downstream cytokines, including caspase-1, IL-1β and IL-18, are highly expressed in the lung tissues of silicosis rats (Song et al., 2018). In a more recent study, this group suggested that inhibiting NLRP3 protein can reduce epithelial–mesenchymal transition of lung macrophages and reduce tissue inflammation (Song et al., 2020). These studies are in line with the hypothesis put forth several years ago that silica induces cell death by stimulating pyroptosis (Reisetter et al., 2011). Interestingly, transplantation of bone marrow mesenchymal stem/stromal cells (BMSCs) attenuated pulmonary fibrosis in a rat model (Li et al., 2018b; Zhang et al., 2018b; Zhao et al., 2021). Furthermore, Zhao et al. were able to demonstrate that the anti-fibrotic effect of transplanted BMSCs in a rat model of silicosis was due to the inhibition of pyroptosis rather than autophagy (Zhao et al., 2021). However, Zhu et al. showed that BMSC transplantation alleviated lung tissue damage in rats by inhibiting autophagy (Zhu et al., 2016). According to Tan et al., these discrepancies can be attributed to differences in the dose and time of administration of BMSCs (Tan and Chen, 2021b). The specific mechanism underlying pyroptosis-dependent progression of silicosis needs to be further explored.

### 4.4 Pyroptosis in liver diseases

Non-alcoholic steatohepatitis (NASH) is a chronic liver disease characterized by immune cell infiltration, intracellular lipid accumulation and hepatocyte ballooning; it is more likely to cause liver fibrosis than simple steatosis, and can lead to liver failure and eventually liver transplantation (Brunt, 2010). The pathogenesis of NASH is complex, and there is no unified conclusion. Steatosis caused by overnutrition, and the resulting insulin resistance, hyperinsulinemia, hyperglycemia, and metabolic syndrome are risk factors for NASH (Cohen et al., 2011; Cusi, 2012; Musso et al., 2012; Farrell et al., 2013; Machado and Diehl, 2016). The interaction of cellular stress factors (oxidation, endoplasmic reticulum and membrane fluidity), signaling pathways [c-Jun N-terminal kinase (JNK) and nuclear factor-κb (NF-κB)] (Farrell et al., 2012), autophagy, cell senescence and mitochondrial damage are the possible mechanisms of NASH (Farrell et al., 2018). With the deepening of academic research on pyroptosis, the role of pyroptosis in the pathogenesis of NASH cannot also be ignored. The receptors of pyroptosis, inflammasomes, are highly expressed in macrophages (Dixon et al., 2012), stellate cells (Watanabe et al., 2009) and hepatocytes (Yan et al., 2012). Rare Earth oxides can activate NLRP3 inflammasome and caspase-1, eventually resulting in pyroptosis of resident macrophages (kc), bone marrow-derived macrophages and other macrophage cell lines such as RAW 264.7 cells (Mirshafiee et al., 2018). The activation of inflammasomes in hepatic stellate cells promotes hepatic fibrosis (Kisseleva & Brenner, 2007). In addition, caspase-1 can also cause hepatic fibrosis and aggravate NASH symptoms in humans (Gaul et al., 2021). Liver cell damage progresses from simple steatosis to NASH to fibrosis, and each process is related to the NLRP3 inflammasome signaling pathway (Mridha et al., 2017). The NLRP3 protein particles emitted by hepatocytes undergoing pyroptosis *in vitro* promoted extracellular matrix production and fibrosis following internalization by the hepatic stellate cells (Gaul et al., 2021). Xu et al. created NASH in WT and Gsdmd-knockout mice by feeding the animals with a methionine- and choline-deficient (MCD) diet, and found that the Gsdmd-knockout mice had significantly attenuated liver inflammatory response and fibrosis in contrast with their WT littermates (Xu et al., 2018b). In addition, the components of the non-canonical inflammatory pathway have also been detected in NASH patients. For instance, Zhu et al. detected a significant increase in caspase-11 levels in the liver tissues of MCD-diet-fed mice. Furthermore, primary hepatocytes isolated from caspase-11 knockout mice were resistant to LPS-induced pyroptosis *in vitro*. The study concluded that caspase-11 deletion can significantly reduce hepatic inflammation and fibrosis by attenuating the pyroptotic response (Zhu et al., 2021).

Alcoholic liver disease (ALD) is induced by perennial alcohol consumption, and it includes alcoholic steatohepatitis (ASH), liver fibrosis, liver cirrhosis and even severe alcoholic hepatitis (AH) that often culminates in liver failure. Liver tissue inflammation in ASH is triggered by the exposure of immune cells to exogenous and endogenous stimuli. The exogenous factors include intestinal microorganisms, whereas the mechanism of aseptic inflammation caused by endogenous factors is still unclear. The inflammasome is a major activator of sterile inflammation and often triggers mitochondrial dysfunction and aberrant purine nucleotide metabolism (Hoek et al., 2002). Furthermore, alcoholic individuals show aberrant mitochondrial function and nucleotide metabolism in the liver tissues, which lead to altered ATP/ADP ratios and uric acid accumulation (Nakamura et al., 2012). Iracheta et al. demonstrated that uric acid and ATP can activate the inflammasome in ASH, prompt IL-1β maturity, and further exacerbate liver inflammation (Iracheta-Vellve et al., 2015). Similarly, Petrasek et al. observed that hepatocytes damaged by alcohol release ATP and uric acid, which result in the release of IL-1β from immune cells (Petrasek et al., 2015). The same group also demonstrated that the NLRP3/caspase-1/IL-1β signaling axis acts as a key character in the progression of steatosis, the inflammatory response and cellular injury in ALD, and that inhibiting the IL-1 signaling pathway alleviated these symptoms (Petrasek et al., 2012). However, a recent study by Khanova et al. showed that pyroptosis in liver inflammation is induced by the non-canonical caspase-11 pathway, rather than caspase-1, and is a key mechanism driving the progression of ASH to AH (Khanova et al., 2018). Pyroptosis is also a vital feature of aseptic liver diseases, such as high fat diet-induced liver damage, polystyrene microplastics-induced liver injury, acetaminophen-induced liver damage, hepatic ischemia–reperfusion damage and cholestatic liver diseases (Zhang et al., 2021c; Lu et al., 2021; Wang et al., 2022b; Han et al., 2022; Mu et al., 2022). Taken together, identification of novel pyroptosis-related targets can provide new insights for the treatment of the above diseases.

### 4.5 Pyroptosis in ocular diseases

Glaucoma is a common degenerative eye disease with an underlying inflammatory basis (Baudouin et al., 2021). Inflammation occurs primarily in the optic nerve head tissue and retina tissue, which show an accumulation of activated astrocytes and microglia, along with high levels of IL-1β (Bordone et al., 2017; Williams et al., 2017). These inflammatory factors further promote oxidative stress, which creates a vicious cycle and eventually results in chronic inflammation (Adornetto et al., 2019; Fernandez-Albarral et al., 2021). Markiewicz et al. believed that the NLRP3 protein is activated in glaucoma patients (Markiewicz et al., 2015). Furthermore, Chen et al. demonstrated that NLRP12 induced pyroptosis in retinal ganglion cells (RGCs) *in vitro* and *in vivo* via caspase-1-mediated GSDMD cleavage in conjunction with NLRP3 and NLRC4. The secreted IL-1β amplified the pyroptotic response by accelerating CASP8-HIF-1α-mediated NLRP12/NLRP3/NLRC4 activation, which exacerbated tissue inflammation. In addition, knocking out the NLRP12 gene significantly reduced RGC loss and the severity of retinal damage, thus confirming that the activation of NLRP12 results in retinal ischemic damage in acute glaucoma. In conclusion, this study offers new insights into the treatment of glaucoma (Chen et al., 2020a).

Dry eye is a common eye disease caused by insufficient secretion or excessive evaporation of tears. A recent study demonstrated that the NLRP3 interacted with ASC proteins to initiate the activation of caspase-1 and the maturation of IL-1β and IL-18 in a mouse model of dry eye, resulting in GSDMD-driven pyroptosis (Swanson et al., 2019). Similarly, Chen et al. demonstrated that GSDMD-dependent pyroptosis induced by NLRP12 and NLRC4 inflammasomes has a crucial role in the development of dry eye, and the accompanying outflow of IL-33 exacerbates the inflammation of corneal epithelial cells. NLRP12/NLRC4 knockdown, GSDMD knockout, or the neutralization of mature IL-33 can obviously attenuate the damage to corneal epithelial cells, indicating that these molecules could be key targets for dry eye treatment in the future (Chen et al., 2020b).

### 4.6 Pyroptosis in renal diseases

Crystal-induced nephropathy has a complex mechanism and ranges from mild/transient to severe/irreparable. Recent research has demonstrated that the NLRP3/caspase-1/IL-1β axis is involved in the occurrence of oxalate nephropathy and calcium oxalate nephropathy (Knauf et al., 2013; Mulay et al., 2013). The crystals deposited in renal tissues can not only activate the NLRP3 inflammasome, but also induce the release of inflammatory factors. Liu et al. recently demonstrated GSDMD-induced pyroptosis in the renal tubular epithelial cells of a calcium oxalate crystal-induced model of nephropathy (Liu et al., 2020). Similarly, Ding et al. detected a simultaneous increase in IL-1β and GSDMD protein in the renal tubular epithelial cells of glyoxylate-treated mice, which confirms the pathological role of GSDMD-dependent pyroptosis in crystal-induced nephropathy (Ding et al., 2021). Consistent with this, Knauf et al. demonstrated that NLRP3 knockout mice fed with a high-soluble-oxalate diet did not exhibit symptoms of progressive renal failure (Knauf et al., 2013).

### 4.7 Pyroptosis in stroke

Inflammation is a crucial pathological mechanism of cerebral ischemic damage and stroke, a common disease with high mortality worldwide. Recent research has demonstrated that pyroptosis is ubiquitous in ischemic brain tissue. Furthermore, inhibiting the activity of inflammasomes closely related to pyroptosis, such as NLRP1, NLRP3, AIM2 and NLRC4, or caspase-1, can effectively alleviate ischemic injury (Barrington et al., 2017). Fann et al. demonstrated that caspase-11 is activated in mouse ischemic cortical neurons (Fann et al., 2013). However, there is ample evidence suggesting that NLRP3 mainly exists in microglia and endothelial cells, but not in the neurons (Yang et al., 2014; Ismael et al., 2018). In fact, activation of PPRs on microglia results in inflammasome-mediated release of IL-1β and production of TNF. Yang et al. found that cerebral infarction and edema were significantly reduced in NLRP3-knockout mice following middle cerebral artery occlusion (MCAO), and the permeability of the blood–brain barrier was acceptable (Yang et al., 2014). Cao et al. further confirmed that the NLRP1 was elevated in a rat model of MCAO, which resulted in an increase in IL-1β and IL-18 levels (Cao et al., 2020). In conclusion, pyroptosis has a vital role in the pathogenesis of stroke, and pyroptosis inhibitors are a promising therapeutic option.

## 5 Conclusion and outlook

Inflammation is a vital factor in immune responses and can be triggered by pathogenic and non-pathogenic factors. On one hand, inflammation promotes clearance of pathogens, which is conducive to tissue repair. On the other hand, an uncontrolled inflammatory response can lead to the accumulation of neutrophils and macrophages that release large amounts of inflammatory factors, resulting in extensive tissue damage. Inflammation is also the basis of the pathogenesis of many diseases. Although we have largely concentrated on the pathological function of pyroptosis in some sterile inflammatory diseases in this review, there is evidence of its involvement in bacterial inflammation such as sepsis and periodontitis. In the initial stages of sepsis, the innate immune cells engulf and kill pathogens via pyroptosis (Miao et al., 2010; Aachoui et al., 2013). However, uncontrolled pyroptosis in the later stages triggers a systemic inflammatory response that eventually results in organ failure or septic shock (Aglietti and Dueber, 2017; Esquerdo et al., 2017; Pfalzgraff et al., 2017; Pu et al., 2017). Similarly, virulence factors in periodontal tissues induce caspase activation, the cleavage of GSDMD, and the release of IL-1β and IL-18, thereby amplifying tissue inflammation through pyroptosis (Sordi et al., 2021).

Furthermore, there is significant crosstalk between pyroptosis and other forms of cell death. One study demonstrated that caspase-1 can activate the pyroptotic pathway in GSDMD-deficient or low-expressing cells by inducing cleavage of the key apoptosis factor, caspase-3 (Taabazuing et al., 2017; Tsuchiya et al., 2019). Another study demonstrated that the apoptosis initiator, capase-8, can also cleave GSDMD, resulting in pyroptosis (Orning et al., 2018; Sarhan et al., 2018; Demarco et al., 2020). Futhermore, apoptotic caspase activity has been demonstrated in wild-type cells in response to various pyroptotic stimuli (Sagulenko et al., 2013), and caspase-3 can cause tissue damage during chemotherapy by inducing pyroptosis via GSDME cleavage (Rogers et al., 2017; Wang et al., 2017). Xu et al. further demonstrated that APAF1 apoptotic bodies can not only activate apoptosis, but also interact with caspase-11 to activate cleavage of caspase-3 and induce GSDME-mediated pyroptosis (Xu et al., 2021). In conclusion, pyroptosis and apoptosis are closely related and mutually regulated at different levels of their respective pathways.

**FIGURE 4:**
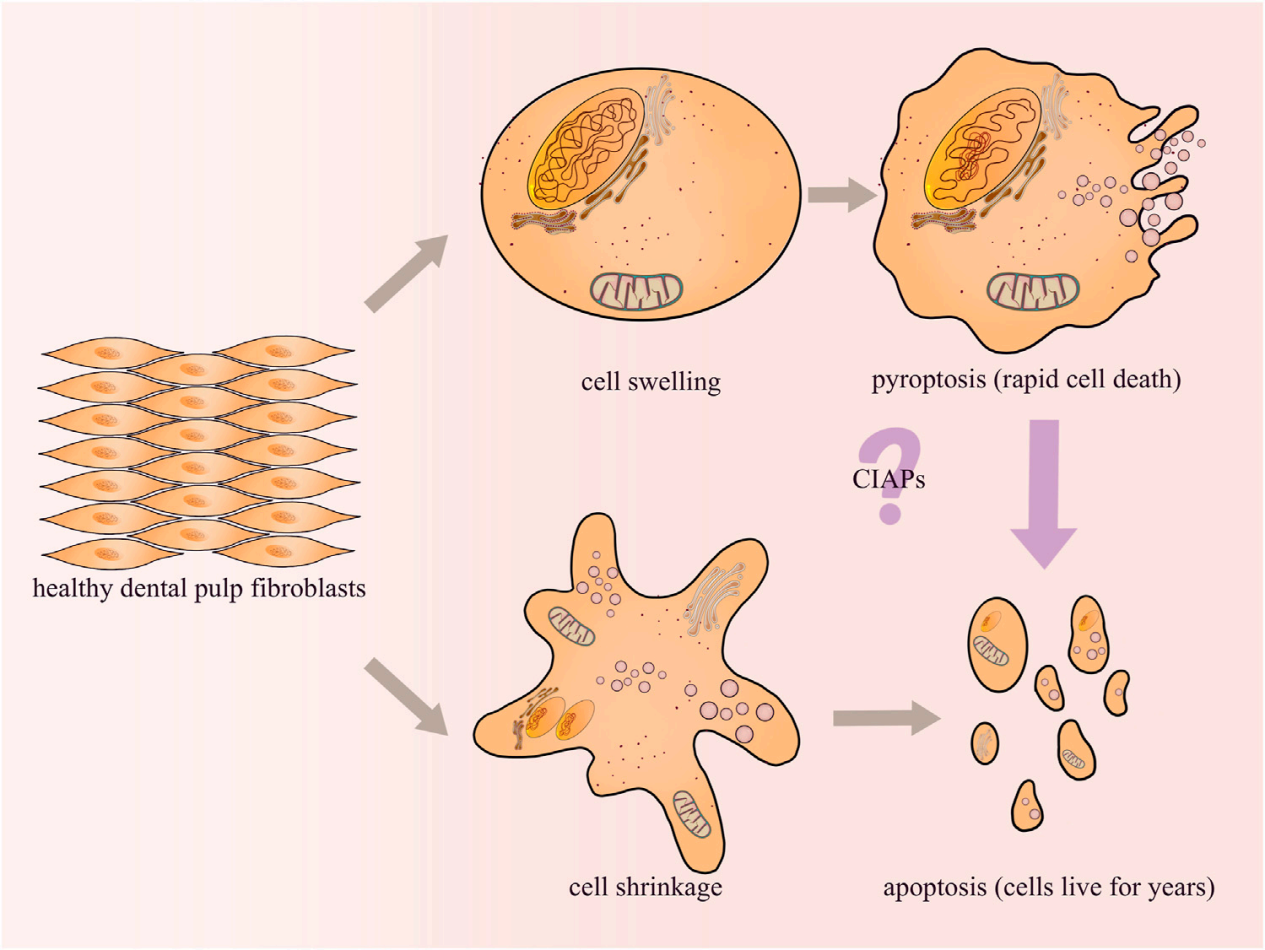
Pulp inflammation may cause pyroptosis and/or apoptosis of DPFs. However, different forms of cell death will affect the survival of the pulp tissue. Our research group will attempt to explore the mechanisms about the crosstalk between pyroptosis and apoptosis in future studies.

In clinical work, it has been observed that the pulp tissue will die through a chronic silent state or an acute state after exposure to cariogenic bacteria infection, traumatic injury or filling material stimulation. The acute state lasts only a few days, while the chronic state lasts for years or even decades before the pulp cells die completely. Previous reports have proved that odontoblasts or dental pulp cells may undergo apoptosis during the progression of dental pulp lesions (Nilsson et al., 2010). Apoptosis has been considered as a highly coordinated, immunologically inert (silent), and non-dramatic process of programmed cell death (Bertheloot et al., 2021). We hypothesized that dental pulp tissue could survive for decades until complete death due to a state of apoptosis. Furthermore, in our studies on the mechanism of pulpitis, we demonstrated that AIM2 and NLRP3 inflammasomes are highly expressed in inflamed pulp tissues (Wang et al., 2013; Jiang et al., 2015). Both AIM2 and NLRP3 can activate caspase-1 and promote the release of IL-1β in dental pulp fibroblasts (DPFs) (Wang et al., 2013; Zhang et al., 2015). Since caspase-1 activation is a key event in the classical pyroptosis signaling pathway, it is reasonable to speculate that pyroptosis may play an important role in the process of acute inflammation in dental pulp. In an inflammatory environment, the pyroptosis of DPFs causes the pulp tissue to die rapidly through an acute state, while the apoptosis of DPFs causes the pulp tissue to survive for years or even decades until death through a chronic state. Studies increasingly demonstrate the complex crosstalk between pyroptosis and apoptosis, and there may be some “switching molecules”. For example, inhibitor of apoptosis proteins (CIAPs), which directly bind to Casp3/7/8/9 and inhibit apoptosis, can also trigger pyroptosis by promoting caspase-1 activation (Feoktistova et al., 2020) (Figure 4). Currently, our research is focusing on this area, and the identification of such molecules will probably offer new insights into endodontic treatment.

## References

[B1] Aachoui Y. Leaf I. A. Hagar J. A. Fontana M. F. Campos C. G. Zak D. E. (2013). Caspase-11 protects against bacteria that escape the vacuole. Science 339 (6122), 975–978. 10.1126/science.1230751 23348507 PMC3697099

[B2] Adornetto A. Russo R. Parisi V. (2019). Neuroinflammation as a target for glaucoma therapy. Neural Regen. Res. 14 (3), 391–394. 10.4103/1673-5374.245465 30539803 PMC6334605

[B3] Aglietti R. A. Dueber E. C. (2017). Recent insights into the molecular mechanisms underlying pyroptosis and gasdermin family functions. Trends Immunol. 38 (4), 261–271. 10.1016/j.it.2017.01.003 28196749

[B4] An X. Zhang Y. Cao Y. Chen J. Qin H. Yang L. (2020). Punicalagin protects diabetic nephropathy by inhibiting pyroptosis based on TXNIP/NLRP3 pathway. Nutrients 12 (5), 1516. 10.3390/nu12051516 32456088 PMC7284711

[B5] Babel R. A. Dandekar M. P. (2021). A review on cellular and molecular mechanisms linked to the development of diabetes complications. Curr. Diabetes Rev. 17 (4), 457–473. 10.2174/1573399816666201103143818 33143626

[B6] Baroja-Mazo A. Martin-Sanchez F. Gomez A. I. Martinez C. M. Amores-Iniesta J. Compan V. (2014). The NLRP3 inflammasome is released as a particulate danger signal that amplifies the inflammatory response. Nat. Immunol. 15 (8), 738–748. 10.1038/ni.2919 24952504

[B7] Barrington J. Lemarchand E. Allan S. M. (2017). A brain in flame; do inflammasomes and pyroptosis influence stroke pathology? Brain Pathol. 27 (2), 205–212. 10.1111/bpa.12476 27997059 PMC8028888

[B8] Baudouin C. Kolko M. Melik-Parsadaniantz S. Messmer E. M. (2021). Inflammation in Glaucoma: From the back to the front of the eye, and beyond. Prog. Retin Eye Res. 83, 100916. 10.1016/j.preteyeres.2020.100916 33075485

[B9] Ben-Chetrit E. Gattorno M. Gul A. Kastner D. L. Lachmann H. J. Touitou I. (2018). Consensus proposal for taxonomy and definition of the autoinflammatory diseases (AIDs): A delphi study. Ann. Rheum. Dis. 77(11), 1558–1565. 10.1136/annrheumdis-2017-212515 30100561

[B10] Bertheloot D. Latz E. Franklin B. S. (2021). Necroptosis, pyroptosis and apoptosis: An intricate game of cell death. Cell Mol. Immunol. 18 (5), 1106–1121. 10.1038/s41423-020-00630-3 33785842 PMC8008022

[B11] Bordone M. P. Gonzalez Fleitas M. F. Pasquini L. A. Bosco A. Sande P. H. Rosenstein R. E. (2017). Involvement of microglia in early axoglial alterations of the optic nerve induced by experimental glaucoma. J. Neurochem. 142 (2), 323–337. 10.1111/jnc.14070 28498493

[B12] Brunt E. M. (2010). Pathology of nonalcoholic fatty liver disease. Nat. Rev. Gastroenterol. Hepatol. 7 (4), 195–203. 10.1038/nrgastro.2010.21 20195271

[B13] Brydges S. D. Broderick L. McGeough M. D. Pena C. A. Mueller J. L. Hoffman H. M. (2013). Divergence of IL-1, IL-18, and cell death in NLRP3 inflammasomopathies. J. Clin. Invest. 123 (11), 4695–4705. 10.1172/JCI71543 24084736 PMC3809806

[B14] Bulek K. Zhao J. Liao Y. Rana N. Corridoni D. Antanaviciute A. (2020). Epithelial-derived gasdermin D mediates nonlytic IL-1β release during experimental colitis. J. Clin. Invest. 130 (8), 4218–4234. 10.1172/JCI138103 32597834 PMC7410065

[B15] Canna S. W. de Jesus A. A. Gouni S. Brooks S. R. Marrero B. Liu Y. (2014). An activating NLRC4 inflammasome mutation causes autoinflammation with recurrent macrophage activation syndrome. Nat. Genet. 46 (10), 1140–1146. 10.1038/ng.3089 25217959 PMC4177369

[B16] Canna S. W. Girard C. Malle L. de Jesus A. Romberg N. Kelsen J. (2017). Life-threatening NLRC4-associated hyperinflammation successfully treated with IL-18 inhibition. J. Allergy Clin. Immunol. 139 (5), 1698–1701. 10.1016/j.jaci.2016.10.022 27876626 PMC5846100

[B17] Cao Y. Zhang H. Lu X. Wang J. Zhang X. Sun S. (2020). Overexpression of MicroRNA-9a-5p ameliorates NLRP1 inflammasome-mediated ischemic injury in rats following ischemic stroke. Neuroscience 444, 106–117. 10.1016/j.neuroscience.2020.01.008 31954830

[B18] Chang H. Chang H. Cheng T. Lee G. D. Chen X. Qi K. (2021). Micro-ribonucleic acid-23a-3p prevents the onset of type 2 diabetes mellitus by suppressing the activation of nucleotide-binding oligomerization-like receptor family pyrin domain containing 3 inflammatory bodies-caused pyroptosis through negatively regulating NIMA-related kinase 7. J. Diabetes Investig. 12 (3), 334–345. 10.1111/jdi.13396 PMC7926233 32881354

[B19] Chao K. L. Kulakova L. Herzberg O. (2017). Gene polymorphism linked to increased asthma and IBD risk alters gasdermin-B structure, a sulfatide and phosphoinositide binding protein. Proc. Natl. Acad. Sci. U. S. A. 114 (7), E1128–E1137. 10.1073/pnas.1616783114 28154144 PMC5321033

[B20] Chen H. Deng Y. Gan X. Li Y. Huang W. Lu L. (2020a). NLRP12 collaborates with NLRP3 and NLRC4 to promote pyroptosis inducing ganglion cell death of acute glaucoma. Mol. Neurodegener. 15 (1), 26. 10.1186/s13024-020-00372-w 32295623 PMC7161290

[B21] Chen H. Gan X. Li Y. Gu J. Liu Y. Deng Y. (2020b). NLRP12- and NLRC4-mediated corneal epithelial pyroptosis is driven by GSDMD cleavage accompanied by IL-33 processing in dry eye. Ocul. Surf. 18 (4), 783–794. 10.1016/j.jtos.2020.07.001 32735949

[B22] Chen Q. Shi P. Wang Y. Zou D. Wu X. Wang D. (2019). GSDMB promotes non-canonical pyroptosis by enhancing caspase-4 activity. J. Mol. Cell Biol. 11 (6), 496–508. 10.1093/jmcb/mjy056 30321352 PMC6734491

[B23] Clough J. N. Omer O. S. Tasker S. Lord G. M. Irving P. M. (2020). Regulatory T-cell therapy in crohn's disease: Challenges and advances. Gut 69 (5), 942–952. 10.1136/gutjnl-2019-319850 31980447 PMC7229901

[B24] Cohen J. C. Horton J. D. Hobbs H. H. (2011). Human fatty liver disease: Old questions and new insights. Science 332 (6037), 1519–1523. 10.1126/science.1204265 21700865 PMC3229276

[B25] Coll R. C. Robertson A. A. Chae J. J. Higgins S. C. Munoz-Planillo R. Inserra M. C. (2015). A small-molecule inhibitor of the NLRP3 inflammasome for the treatment of inflammatory diseases. Nat. Med. 21 (3), 248–255. 10.1038/nm.3806 25686105 PMC4392179

[B26] Cookson B. T. Brennan M. A. (2001). Pro-inflammatory programmed cell death. Trends Microbiol. 9 (3), 113–114. 10.1016/s0966-842x(00)01936-3 11303500

[B27] Cusi K. (2012). Role of obesity and lipotoxicity in the development of nonalcoholic steatohepatitis: Pathophysiology and clinical implications. Gastroenterology 142 (4), 711–725. 10.1053/j.gastro.2012.02.003 22326434

[B28] Cypryk W. Nyman T. A. Matikainen S. (2018). From inflammasome to exosome-does extracellular vesicle secretion constitute an inflammasome-dependent immune response? Front. Immunol. 9, 2188. 10.3389/fimmu.2018.02188 30319640 PMC6167409

[B29] Dai X. Sun F. Deng K. Lin G. Yin W. Chen H. (2022). Mallotucin D, a clerodane diterpenoid from Croton crassifolius, suppresses HepG2 cell growth via inducing autophagic cell death and pyroptosis. Int. J. Mol. Sci. 23 (22), 14217. 10.3390/ijms232214217 36430694 PMC9698996

[B30] Demarco B. Grayczyk J. P. Bjanes E. Le Roy D. Tonnus W. Assenmacher C. A. (2020). Caspase-8-dependent gasdermin D cleavage promotes antimicrobial defense but confers susceptibility to TNF-induced lethality. Sci. Adv. 6 (47), eabc3465. 10.1126/sciadv.abc3465 33208362 PMC7673803

[B31] Demircan C. Akdogan N. Elmas L. (2020). Nicolau syndrome secondary to subcutaneous glatiramer acetate injection. Int. J. Low. Extrem Wounds 22, 149–151. 10.1177/1534734620973144 33258397

[B32] Deng W. Bai Y. Deng F. Pan Y. Mei S. Zheng Z. (2022). Streptococcal pyrogenic exotoxin B cleaves GSDMA and triggers pyroptosis. Nature 602 (7897), 496–502. 10.1038/s41586-021-04384-4 35110732 PMC9703647

[B33] Ding J. Wang K. Liu W. She Y. Sun Q. Shi J. (2016). Pore-forming activity and structural autoinhibition of the gasdermin family. Nature 535 (7610), 111–116. 10.1038/nature18590 27281216

[B34] Ding T. Zhao T. Li Y. Liu Z. Ding J. Ji B. (2021). Vitexin exerts protective effects against calcium oxalate crystal-induced kidney pyroptosis *in vivo* and *in vitro* . Phytomedicine 86, 153562. 10.1016/j.phymed.2021.153562 33857849

[B35] Dixon L. J. Berk M. Thapaliya S. Papouchado B. G. Feldstein A. E. (2012). Caspase-1-mediated regulation of fibrogenesis in diet-induced steatohepatitis. Lab. Invest. 92 (5), 713–723. 10.1038/labinvest.2012.45 22411067 PMC3808241

[B36] Dutta P. Courties G. Wei Y. Leuschner F. Gorbatov R. Robbins C. S. (2012). Myocardial infarction accelerates atherosclerosis. Nature 487 (7407), 325–329. 10.1038/nature11260 22763456 PMC3401326

[B37] Esquerdo K. F. Sharma N. K. Brunialti M. K. C. Baggio-Zappia G. L. Assuncao M. Azevedo L. C. P. (2017). Inflammasome gene profile is modulated in septic patients, with a greater magnitude in non-survivors. Clin. Exp. Immunol. 189 (2), 232–240. 10.1111/cei.12971 28369745 PMC5508322

[B38] Evavold C. L. Ruan J. Tan Y. Xia S. Wu H. Kagan J. C. (2018). The pore-forming protein gasdermin D regulates interleukin-1 secretion from living macrophages. Immunity 48 (1), 35–44. 10.1016/j.immuni.2017.11.013 29195811 PMC5773350

[B39] Fang Y. Tian S. Pan Y. Li W. Wang Q. Tang Y. (2020). Pyroptosis: A new frontier in cancer. Biomed. Pharmacother. 121, 109595. 10.1016/j.biopha.2019.109595 31710896

[B40] Fann D. Y. Lee S. Y. Manzanero S. Tang S. C. Gelderblom M. Chunduri P. (2013). Intravenous immunoglobulin suppresses NLRP1 and NLRP3 inflammasome-mediated neuronal death in ischemic stroke. Cell Death Dis. 4 (9), e790. 10.1038/cddis.2013.326 24008734 PMC3789184

[B41] Farrell G. C. Haczeyni F. Chitturi S. (2018). Pathogenesis of NASH: How metabolic complications of overnutrition favour lipotoxicity and pro-inflammatory fatty liver disease. Adv. Exp. Med. Biol. 1061, 19–44. 10.1007/978-981-10-8684-7_3 29956204

[B42] Farrell G. C. McCullough A. J. Day C. P. (2013). Non-alcoholic fatty liver disease: A practical guide. Netherlands: Wiley-Blackwell.

[B43] Farrell G. C. van Rooyen D. Gan L. Chitturi S. (2012). NASH is an inflammatory disorder: Pathogenic, prognostic and therapeutic implications. Gut Liver 6 (2), 149–171. 10.5009/gnl.2012.6.2.149 22570745 PMC3343154

[B44] Feoktistova M. Makarov R. Brenji S. Schneider A. T. Hooiveld G. J. Luedde T. (2020). A20 promotes ripoptosome formation and TNF-induced apoptosis via cIAPs regulation and NIK stabilization in keratinocytes. Cells 9 (2), 351. 10.3390/cells9020351 32028675 PMC7072579

[B45] Fernandez-Albarral J. A. Salazar J. J. de Hoz R. Marco E. M. Martin-Sanchez B. Flores-Salguero E. (2021). Retinal molecular changes are associated with neuroinflammation and loss of RGCs in an experimental model of glaucoma. Int. J. Mol. Sci. 22 (4), 2066. 10.3390/ijms22042066 33669765 PMC7922243

[B46] Franchi L. Amer A. Body-Malapel M. Kanneganti T. D. Ozoren N. Jagirdar R. (2006). Cytosolic flagellin requires Ipaf for activation of caspase-1 and interleukin 1beta in salmonella-infected macrophages. Nat. Immunol. 7 (6), 576–582. 10.1038/ni1346 16648852

[B47] Franklin B. S. Bossaller L. De Nardo D. Ratter J. M. Stutz A. Engels G. (2014). The adaptor ASC has extracellular and 'prionoid' activities that propagate inflammation. Nat. Immunol. 15 (8), 727–737. 10.1038/ni.2913 24952505 PMC4116676

[B48] Gan J. Huang M. Lan G. Liu L. Xu F. (2020). High glucose induces the loss of retinal pericytes partly via NLRP3-caspase-1-GSDMD-mediated pyroptosis. Biomed. Res. Int. 2020, 4510628. 10.1155/2020/4510628 32420343 PMC7201508

[B49] Gao W. Yang J. Liu W. Wang Y. Shao F. (2016). Site-specific phosphorylation and microtubule dynamics control Pyrin inflammasome activation. Proc. Natl. Acad. Sci. U. S. A. 113 (33), E4857–E4866. 10.1073/pnas.1601700113 27482109 PMC4995971

[B50] Gaul S. Leszczynska A. Alegre F. Kaufmann B. Johnson C. D. Adams L. A. (2021). Hepatocyte pyroptosis and release of inflammasome particles induce stellate cell activation and liver fibrosis. J. Hepatol. 74 (1), 156–167. 10.1016/j.jhep.2020.07.041 32763266 PMC7749849

[B51] Gil-Lianes J. Gariup G. Iranzo-Fernandez P. Mensa-Vilaro A. Penin-Franch A. Hurtado-Navarro L. (2023). Early-onset recurrent panniculitis as a phenotype of NLRC4-associated autoinflammatory syndrome: Characterization of pathogenicity of the p.Ser445Pro NLRC4 variant. Australas. J. Dermatol 2023, 14005. 10.1111/ajd.14005 36797819

[B52] Goldfinger S. E. (1972). Colchicine for familial Mediterranean fever. N. Engl. J. Med. 287 (25), 1302. 10.1056/NEJM197212212872514 4636899

[B53] Gong W. Zheng T. Guo K. Fang M. Xie H. Li W. (2020). Mincle/syk signalling promotes intestinal mucosal inflammation through induction of macrophage pyroptosis in crohn's disease. J. Crohns Colitis 14 (12), 1734–1747. 10.1093/ecco-jcc/jjaa088 32333776

[B54] Han D. Kim H. Kim S. Le Q. A. Han S. Y. Bae J. (2022). Sestrin2 protects against cholestatic liver injury by inhibiting endoplasmic reticulum stress and NLRP3 inflammasome-mediated pyroptosis. Exp. Mol. Med. 54 (3), 239–251. 10.1038/s12276-022-00737-9 35260799 PMC8980001

[B55] Han Y. Xu X. Tang C. Gao P. Chen X. Xiong X. (2018). Reactive oxygen species promote tubular injury in diabetic nephropathy: The role of the mitochondrial ros-txnip-nlrp3 biological axis. Redox Biol. 16, 32–46. 10.1016/j.redox.2018.02.013 29475133 PMC5842313

[B56] Hao K. Jiang W. Zhou M. Li H. Chen Y. Jiang F. (2020). Targeting BRD4 prevents acute gouty arthritis by regulating pyroptosis. Int. J. Biol. Sci. 16 (16), 3163–3173. 10.7150/ijbs.46153 33162822 PMC7645998

[B57] Heger J. Schulz R. Euler G. (2016). Molecular switches under TGFβ signalling during progression from cardiac hypertrophy to heart failure. Br. J. Pharmacol. 173 (1), 3–14. 10.1111/bph.13344 26431212 PMC4813390

[B58] Hoek J. B. Cahill A. Pastorino J. G. (2002). Alcohol and mitochondria: A dysfunctional relationship. Gastroenterology 122 (7), 2049–2063. 10.1053/gast.2002.33613 12055609 PMC1868435

[B59] Hong Y. Standing A. S. I. Nanthapisal S. Sebire N. Jolles S. Omoyinmi E. (2019). Autoinflammation due to homozygous S208 MEFV mutation. Ann. Rheum. Dis. 78 (4), 571–573. 10.1136/annrheumdis-2018-214102 30355575 PMC6530076

[B60] Hou J. Kang Y. J. (2012). Regression of pathological cardiac hypertrophy: Signaling pathways and therapeutic targets. Pharmacol. Ther. 135 (3), 337–354. 10.1016/j.pharmthera.2012.06.006 22750195 PMC3458709

[B61] Hou J. Zhao R. Xia W. Chang C. W. You Y. Hsu J. M. (2020). PD-L1-mediated gasdermin C expression switches apoptosis to pyroptosis in cancer cells and facilitates tumour necrosis. Nat. Cell Biol. 22 (10), 1264–1275. 10.1038/s41556-020-0575-z 32929201 PMC7653546

[B62] Hu Q. Zhang T. Yi L. Zhou X. Mi M. (2018). Dihydromyricetin inhibits NLRP3 inflammasome-dependent pyroptosis by activating the Nrf2 signaling pathway in vascular endothelial cells. Biofactors 44 (2), 123–136. 10.1002/biof.1395 29193391

[B63] Hu Z. Murakami T. Suzuki K. Tamura H. Kuwahara-Arai K. Iba T. (2014). Antimicrobial cathelicidin peptide LL-37 inhibits the LPS/ATP-induced pyroptosis of macrophages by dual mechanism. PLoS One 9 (1), e85765. 10.1371/journal.pone.0085765 24454930 PMC3894207

[B64] Iannantuoni F. Diaz-Morales N. Escribano-Lopez I. Sola E. Roldan-Torres I. Apostolova N. (2019). Does glycemic control modulate the impairment of NLRP3 inflammasome activation in type 2 diabetes? Antioxid. Redox Signal 30 (2), 232–240. 10.1089/ars.2018.7582 29860862

[B65] Iracheta-Vellve A. Petrasek J. Satishchandran A. Gyongyosi B. Saha B. Kodys K. (2015). Inhibition of sterile danger signals, uric acid and ATP, prevents inflammasome activation and protects from alcoholic steatohepatitis in mice. J. Hepatol. 63 (5), 1147–1155. 10.1016/j.jhep.2015.06.013 26100496 PMC4615393

[B66] Ismael S. Zhao L. Nasoohi S. Ishrat T. (2018). Inhibition of the NLRP3-inflammasome as a potential approach for neuroprotection after stroke. Sci. Rep. 8 (1), 5971. 10.1038/s41598-018-24350-x 29654318 PMC5899150

[B67] Jha J. C. Banal C. Chow B. S. Cooper M. E. Jandeleit-Dahm K. (2016). Diabetes and kidney disease: Role of oxidative stress. Antioxid. Redox Signal 25 (12), 657–684. 10.1089/ars.2016.6664 26906673 PMC5069735

[B68] Jia C. Zhang J. Chen H. Zhuge Y. Chen H. Qian F. (2019). Endothelial cell pyroptosis plays an important role in Kawasaki disease via HMGB1/RAGE/cathespin B signaling pathway and NLRP3 inflammasome activation. Cell Death Dis. 10 (10), 778. 10.1038/s41419-019-2021-3 31611559 PMC6791856

[B69] Jiang W. Lv H. Wang H. Wang D. Sun S. Jia Q. (2015). Activation of the NLRP3/caspase-1 inflammasome in human dental pulp tissue and human dental pulp fibroblasts. Cell Tissue Res. 361 (2), 541–555. 10.1007/s00441-015-2118-7 25684031 PMC4529451

[B70] Joshi G. N. Knecht D. A. (2013). Silica phagocytosis causes apoptosis and necrosis by different temporal and molecular pathways in alveolar macrophages. Apoptosis 18 (3), 271–285. 10.1007/s10495-012-0798-y 23329178

[B71] Kang M. J. Yu H. S. Seo J. H. Kim H. Y. Jung Y. H. Kim Y. J. (2012). GSDMB/ORMDL3 variants contribute to asthma susceptibility and eosinophil-mediated bronchial hyperresponsiveness. Hum. Immunol. 73 (9), 954–959. 10.1016/j.humimm.2012.06.009 22732088

[B72] Kayagaki N. Stowe I. B. Lee B. L. O'Rourke K. Anderson K. Warming S. (2015). Caspase-11 cleaves gasdermin D for non-canonical inflammasome signalling. Nature 526 (7575), 666–671. 10.1038/nature15541 26375259

[B73] Kehlet S. N. Willumsen N. Armbrecht G. Dietzel R. Brix S. Henriksen K. (2018). Age-related collagen turnover of the interstitial matrix and basement membrane: Implications of age- and sex-dependent remodeling of the extracellular matrix. PLoS One 13 (3), e0194458. 10.1371/journal.pone.0194458 29596429 PMC5875766

[B74] Khanova E. Wu R. Wang W. Yan R. Chen Y. French S. W. (2018). Pyroptosis by caspase11/4-gasdermin-D pathway in alcoholic hepatitis in mice and patients. Hepatology 67 (5), 1737–1753. 10.1002/hep.29645 29108122 PMC5906140

[B75] Kisseleva T. Brenner D. A. (2007). Role of hepatic stellate cells in fibrogenesis and the reversal of fibrosis. J. Gastroenterol. Hepatol. 22 (1), S73–S78. 10.1111/j.1440-1746.2006.04658.x 17567473

[B76] Knauf F. Asplin J. R. Granja I. Schmidt I. M. Moeckel G. W. David R. J. (2013). NALP3-mediated inflammation is a principal cause of progressive renal failure in oxalate nephropathy. Kidney Int. 84 (5), 895–901. 10.1038/ki.2013.207 23739234 PMC3772982

[B77] LaRock D. L. Johnson A. F. Wilde S. Sands J. S. Monteiro M. P. LaRock C. N. (2022). Group A Streptococcus induces GSDMA-dependent pyroptosis in keratinocytes. Nature 605 (7910), 527–531. 10.1038/s41586-022-04717-x 35545676 PMC9186297

[B78] Lee J. Wan J. Lee L. Peng C. Xie H. Lee C. (2017). Study of the NLRP3 inflammasome component genes and downstream cytokines in patients with type 2 diabetes mellitus with carotid atherosclerosis. Lipids Health Dis. 16 (1), 217. 10.1186/s12944-017-0595-2 29151018 PMC5694162

[B79] Li P. An G. Wang Y. Liang D. Zhu Z. Tian L. (2018b). Targeted migration of bone marrow mesenchymal stem cells inhibits silica-induced pulmonary fibrosis in rats. Stem Cell Res. Ther. 9 (1), 335. 10.1186/s13287-018-1083-y 30514375 PMC6280342

[B80] Li P. Zhong X. Li J. Liu H. Ma X. He R. (2018a). MicroRNA-30c-5p inhibits NLRP3 inflammasome-mediated endothelial cell pyroptosis through FOXO3 down-regulation in atherosclerosis. Biochem. Biophys. Res. Commun. 503 (4), 2833–2840. 10.1016/j.bbrc.2018.08.049 30119891

[B81] Li R. Y. Zheng Z. Y. Li Z. M. Heng J. H. Zheng Y. Q. Deng D. X. (2022). Cisplatin-induced pyroptosis is mediated via the CAPN1/CAPN2-BAK/BAX-caspase-9-caspase-3-GSDME axis in esophageal cancer. Chem. Biol. Interact. 361, 109967. 10.1016/j.cbi.2022.109967 35525317

[B82] Liang J. Alfano D. N. Squires J. E. Riley M. M. Parks W. T. Kofler J. (2017). Novel NLRC4 mutation causes a syndrome of perinatal autoinflammation with hemophagocytic lymphohistiocytosis, hepatosplenomegaly, fetal thrombotic vasculopathy, and congenital anemia and ascites. Pediatr. Dev. Pathol. 20 (6), 498–505. 10.1177/1093526616686890 28403691

[B83] Lin X. Wang H. An X. Zhang J. Kuang J. Hou J. (2021). Baeckein E suppressed NLRP3 inflammasome activation through inhibiting both the priming and assembly procedure: Implications for gout therapy. Phytomedicine 84, 153521. 10.1016/j.phymed.2021.153521 33667838

[B84] Lin Y. Hu Y. Hu X. Yang L. Chen X. Li Q. (2020a). Ginsenoside Rb2 improves insulin resistance by inhibiting adipocyte pyroptosis. Adipocyte 9 (1), 302–312. 10.1080/21623945.2020.1778826 32580621 PMC7469678

[B85] Lin Y. Luo T. Weng A. Huang X. Yao Y. Fu Z. (2020b). Gallic acid alleviates gouty arthritis by inhibiting NLRP3 inflammasome activation and pyroptosis through enhancing Nrf2 signaling. Front. Immunol. 11, 580593. 10.3389/fimmu.2020.580593 33365024 PMC7750458

[B86] Liu J. Yang K. Jin Y. Liu Y. Chen Y. Zhang X. (2020). H3 relaxin protects against calcium oxalate crystal-induced renal inflammatory pyroptosis. Cell Prolif. 53 (10), e12902. 10.1111/cpr.12902 32945585 PMC7574868

[B87] Liu Y. Xu Z. Ma F. Jia Y. Wang G. (2018). Knockdown of TLR4 attenuates high glucose-induced podocyte injury via the NALP3/ASC/Caspase-1 signaling pathway. Biomed. Pharmacother. 107, 1393–1401. 10.1016/j.biopha.2018.08.134 30257355

[B88] Lopez-Pastrana J. Ferrer L. M. Li Y. F. Xiong X. Xi H. Cueto R. (2015). Inhibition of caspase-1 activation in endothelial cells improves angiogenesis: A novel therapeutic potential for ischemia. J. Biol. Chem. 290 (28), 17485–17494. 10.1074/jbc.M115.641191 26037927 PMC4498083

[B89] Lu J. Wang X. Feng Z. Chen Y. Wen D. Liu Z. (2021). The protective effect of isoflurane pretreatment on liver IRI by suppressing noncanonical pyroptosis of liver macrophages. Int. Immunopharmacol. 99, 107977. 10.1016/j.intimp.2021.107977 34332342

[B90] Ma C. Yang D. Wang B. Wu C. Wu Y. Li S. (2020). Gasdermin D in macrophages restrains colitis by controlling cGAS-mediated inflammation. Sci. Adv. 6 (21), eaaz6717. 10.1126/sciadv.aaz6717 32671214 PMC7314554

[B91] Machado M. V. Diehl A. M. (2016). Pathogenesis of nonalcoholic steatohepatitis. Gastroenterology 150 (8), 1769–1777. 10.1053/j.gastro.2016.02.066 26928243 PMC4887389

[B92] Magnotti F. Lefeuvre L. Benezech S. Malsot T. Waeckel L. Martin A. (2019). Pyrin dephosphorylation is sufficient to trigger inflammasome activation in familial Mediterranean fever patients. EMBO Mol. Med. 11 (11), e10547. 10.15252/emmm.201910547 31589380 PMC6835204

[B93] Markiewicz L. Pytel D. Mucha B. Szymanek K. Szaflik J. Szaflik J. P. (2015). Altered expression levels of MMP1, MMP9, MMP12, TIMP1, and IL-1β as a risk factor for the elevated IOP and optic nerve head damage in the primary open-angle glaucoma patients. Biomed. Res. Int. 2015, 812503. 10.1155/2015/812503 26120586 PMC4442285

[B94] Martinon F. Burns K. Tschopp J. (2002). The inflammasome: A molecular platform triggering activation of inflammatory caspases and processing of proIL-beta. Mol. Cell 10 (2), 417–426. 10.1016/s1097-2765(02)00599-3 12191486

[B95] Masters S. L. Lagou V. Jeru I. Baker P. J. Van Eyck L. Parry D. A. (2016). Familial autoinflammation with neutrophilic dermatosis reveals a regulatory mechanism of pyrin activation. Sci. Transl. Med. 8 (332), 332ra45. 10.1126/scitranslmed.aaf1471 27030597

[B96] McDermott M. F. Aksentijevich I. Galon J. McDermott E. M. Ogunkolade B. W. Centola M. (1999). Germline mutations in the extracellular domains of the 55 kDa TNF receptor, TNFR1, define a family of dominantly inherited autoinflammatory syndromes. Cell 97 (1), 133–144. 10.1016/s0092-8674(00)80721-7 10199409

[B97] McGrath K. C. Li X. H. McRobb L. S. Heather A. K. Gangoda S. V. S. (2015). Inhibitory effect of a French maritime pine bark extract-based nutritional supplement on TNF-alpha-induced inflammation and oxidative stress in human coronary artery endothelial cells. Evid. Based Complement. Altern. Med. 2015, 260530. 10.1155/2015/260530 PMC4664804 26664450

[B98] Miao E. A. Leaf I. A. Treuting P. M. Mao D. P. Dors M. Sarkar A. (2010). Caspase-1-induced pyroptosis is an innate immune effector mechanism against intracellular bacteria. Nat. Immunol. 11 (12), 1136–1142. 10.1038/ni.1960 21057511 PMC3058225

[B99] Mirshafiee V. Sun B. Chang C. H. Liao Y. P. Jiang W. Jiang J. (2018). Toxicological profiling of metal oxide nanoparticles in liver context reveals pyroptosis in kupffer cells and macrophages versus apoptosis in hepatocytes. ACS Nano 12 (4), 3836–3852. 10.1021/acsnano.8b01086 29543433 PMC5946698

[B100] Mitchell P. S. Sandstrom A. Vance R. E. (2019). The NLRP1 inflammasome: New mechanistic insights and unresolved mysteries. Curr. Opin. Immunol. 60, 37–45. 10.1016/j.coi.2019.04.015 31121538 PMC6800612

[B101] Moffatt M. F. Kabesch M. Liang L. Dixon A. L. Strachan D. Heath S. (2007). Genetic variants regulating ORMDL3 expression contribute to the risk of childhood asthma. Nature 448 (7152), 470–473. 10.1038/nature06014 17611496

[B102] Moghaddas F. Llamas R. De Nardo D. Martinez-Banaclocha H. Martinez-Garcia J. J. Mesa-Del-Castillo P. (2017). A novel Pyrin-Associated Autoinflammation with Neutrophilic Dermatosis mutation further defines 14-3-3 binding of pyrin and distinction to Familial Mediterranean Fever. Ann. Rheum. Dis. 76 (12), 2085–2094. 10.1136/annrheumdis-2017-211473 28835462 PMC5687562

[B103] Morikawa S. Kaneko N. Okumura C. Taguchi H. Kurata M. Yamamoto T. (2018). IAPP/amylin deposition, which is correlated with expressions of ASC and IL-1β in β-cells of Langerhans' islets, directly initiates NLRP3 inflammasome activation. Int. J. Immunopathol. Pharmacol. 32, 2058738418788749. 10.1177/2058738418788749 30014749 PMC6050799

[B104] Mridha A. R. Wree A. Robertson A. A. B. Yeh M. M. Johnson C. D. Van Rooyen D. M. (2017). NLRP3 inflammasome blockade reduces liver inflammation and fibrosis in experimental NASH in mice. J. Hepatol. 66 (5), 1037–1046. 10.1016/j.jhep.2017.01.022 28167322 PMC6536116

[B105] Mu Y. Sun J. Li Z. Zhang W. Liu Z. Li C. (2022). Activation of pyroptosis and ferroptosis is involved in the hepatotoxicity induced by polystyrene microplastics in mice. Chemosphere 291 (2), 132944. 10.1016/j.chemosphere.2021.132944 34793849

[B106] Mulay S. R. Kulkarni O. P. Rupanagudi K. V. Migliorini A. Darisipudi M. N. Vilaysane A. (2013). Calcium oxalate crystals induce renal inflammation by NLRP3-mediated IL-1β secretion. J. Clin. Invest. 123 (1), 236–246. 10.1172/JCI63679 23221343 PMC3533282

[B107] Musso G. Cassader M. Rosina F. Gambino R. (2012). Impact of current treatments on liver disease, glucose metabolism and cardiovascular risk in non-alcoholic fatty liver disease (NAFLD): A systematic review and meta-analysis of randomised trials. Diabetologia 55 (4), 885–904. 10.1007/s00125-011-2446-4 22278337

[B108] Nakagawa I. Amano A. Mizushima N. Yamamoto A. Yamaguchi H. Kamimoto T. (2004). Autophagy defends cells against invading group A Streptococcus. Science 306 (5698), 1037–1040. 10.1126/science.1103966 15528445

[B109] Nakamura K. Sakurai M. Miura K. Morikawa Y. Yoshita K. Ishizaki M. (2012). Alcohol intake and the risk of hyperuricaemia: A 6-year prospective study in Japanese men. Nutr. Metab. Cardiovasc Dis. 22 (11), 989–996. 10.1016/j.numecd.2011.01.003 21421297

[B110] Nilsson B. Korsgren O. Lambris J. D. Ekdahl K. N. (2010). Can cells and biomaterials in therapeutic medicine be shielded from innate immune recognition? Trends Immunol. 31 (1), 32–38. 10.1016/j.it.2009.09.005 19836998 PMC2818156

[B111] Oh S. Son M. Park C. H. Jang J. T. Son K. H. Byun K. (2020). The reducing effects of pyrogallol-phloroglucinol-6,6-bieckol on high-fat diet-induced pyroptosis in endothelial and vascular smooth muscle cells of mice aortas. Mar. Drugs 18 (12), 648. 10.3390/md18120648 33339328 PMC7766911

[B112] Oltra S. S. Colomo S. Sin L. Perez-Lopez M. Lazaro S. Molina-Crespo A. (2023). Distinct GSDMB protein isoforms and protease cleavage processes differentially control pyroptotic cell death and mitochondrial damage in cancer cells. Cell Death Differ. 23, 01143. 10.1038/s41418-023-01143-y PMC10154425 36899106

[B113] Orning P. Weng D. Starheim K. Ratner D. Best Z. Lee B. (2018). Pathogen blockade of TAK1 triggers caspase-8-dependent cleavage of gasdermin D and cell death. Science 362 (6418), 1064–1069. 10.1126/science.aau2818 30361383 PMC6522129

[B114] Panganiban R. A. Sun M. Dahlin A. Park H. R. Kan M. Himes B. E. (2018). A functional splice variant associated with decreased asthma risk abolishes the ability of gasdermin B to induce epithelial cell pyroptosis. J. Allergy Clin. Immunol. 142 (5), 1469–1478. 10.1016/j.jaci.2017.11.040 29330013 PMC6037620

[B115] Papin S. Duquesnoy P. Cazeneuve C. Pantel J. Coppey-Moisan M. Dargemont C. (2000). Alternative splicing at the MEFV locus involved in familial Mediterranean fever regulates translocation of the marenostrin/pyrin protein to the nucleus. Hum. Mol. Genet. 9 (20), 3001–3009. 10.1093/hmg/9.20.3001 11115844

[B116] Petrasek J. Bala S. Csak T. Lippai D. Kodys K. Menashy V. (2012). IL-1 receptor antagonist ameliorates inflammasome-dependent alcoholic steatohepatitis in mice. J. Clin. Invest. 122 (10), 3476–3489. 10.1172/JCI60777 22945633 PMC3461900

[B117] Petrasek J. Iracheta-Vellve A. Saha B. Satishchandran A. Kodys K. Fitzgerald K. A. (2015). Metabolic danger signals, uric acid and ATP, mediate inflammatory cross-talk between hepatocytes and immune cells in alcoholic liver disease. J. Leukoc. Biol. 98 (2), 249–256. 10.1189/jlb.3AB1214-590R 25934928 PMC4501673

[B118] Pfalzgraff A. Heinbockel L. Su Q. Brandenburg K. Weindl G. (2017). Synthetic anti-endotoxin peptides inhibit cytoplasmic LPS-mediated responses. Biochem. Pharmacol. 140, 64–72. 10.1016/j.bcp.2017.05.015 28539262

[B119] Platnich J. M. Muruve D. A. (2019). NOD-like receptors and inflammasomes: A review of their canonical and non-canonical signaling pathways. Arch. Biochem. Biophys. 670, 4–14. 10.1016/j.abb.2019.02.008 30772258

[B120] Pu Q. Gan C. Li R. Li Y. Tan S. Li X. (2017). Atg7 deficiency intensifies inflammasome activation and pyroptosis in Pseudomonas sepsis. J. Immunol. 198 (8), 3205–3213. 10.4049/jimmunol.1601196 28258192 PMC5382979

[B121] Quan B. Zhang H. Xue R. (2019). Retracted: miR-141 alleviates LPS-induced inflammation injury in WI-38 fibroblasts by up-regulation of NOX2. Life Sci. 216, 271–278. 10.1016/j.lfs.2018.11.056 30500550

[B122] Rathinam V. A. Vanaja S. K. Fitzgerald K. A. (2012). Regulation of inflammasome signaling. Nat. Immunol. 13 (4), 333–342. 10.1038/ni.2237 22430786 PMC3523703

[B123] Rayamajhi M. Zak D. E. Chavarria-Smith J. Vance R. E. Miao E. A. (2013). Cutting edge: Mouse NAIP1 detects the type III secretion system needle protein. J. Immunol. 191 (8), 3986–3989. 10.4049/jimmunol.1301549 24043898 PMC3819181

[B124] Reisetter A. C. Stebounova L. V. Baltrusaitis J. Powers L. Gupta A. Grassian V. H. (2011). Induction of inflammasome-dependent pyroptosis by carbon black nanoparticles. J. Biol. Chem. 286 (24), 21844–21852. 10.1074/jbc.M111.238519 21525001 PMC3122239

[B125] Rogers C. Fernandes-Alnemri T. Mayes L. Alnemri D. Cingolani G. Alnemri E. S. (2017). Cleavage of DFNA5 by caspase-3 during apoptosis mediates progression to secondary necrotic/pyroptotic cell death. Nat. Commun. 8, 14128. 10.1038/ncomms14128 28045099 PMC5216131

[B126] Romberg N. Al Moussawi K. Nelson-Williams C. Stiegler A. L. Loring E. Choi M. (2014). Mutation of NLRC4 causes a syndrome of enterocolitis and autoinflammation. Nat. Genet. 46 (10), 1135–1139. 10.1038/ng.3066 25217960 PMC4177367

[B127] Sagulenko V. Thygesen S. J. Sester D. P. Idris A. Cridland J. A. Vajjhala P. R. (2013). AIM2 and NLRP3 inflammasomes activate both apoptotic and pyroptotic death pathways via ASC. Cell Death Differ. 20 (9), 1149–1160. 10.1038/cdd.2013.37 23645208 PMC3741496

[B128] Sarhan J. Liu B. C. Muendlein H. I. Li P. Nilson R. Tang A. Y. (2018). Caspase-8 induces cleavage of gasdermin D to elicit pyroptosis during Yersinia infection. Proc. Natl. Acad. Sci. U. S. A. 115 (46), E10888–E10897. 10.1073/pnas.1809548115 30381458 PMC6243247

[B129] Semino C. Carta S. Gattorno M. Sitia R. Rubartelli A. (2018). Progressive waves of IL-1β release by primary human monocytes via sequential activation of vesicular and gasdermin D-mediated secretory pathways. Cell Death Dis. 9 (11), 1088. 10.1038/s41419-018-1121-9 30352992 PMC6199333

[B130] Shahzad K. Bock F. Al-Dabet M. M. Gadi I. Kohli S. Nazir S. (2016). Caspase-1, but not caspase-3, promotes diabetic nephropathy. J. Am. Soc. Nephrol. 27 (8), 2270–2275. 10.1681/ASN.2015060676 26832955 PMC4978051

[B131] Shahzad K. Bock F. Dong W. Wang H. Kopf S. Kohli S. (2015). Nlrp3-inflammasome activation in non-myeloid-derived cells aggravates diabetic nephropathy. Kidney Int. 87 (1), 74–84. 10.1038/ki.2014.271 25075770 PMC4284813

[B132] Shi J. Zhao Y. Wang K. Shi X. Wang Y. Huang H. (2015). Cleavage of GSDMD by inflammatory caspases determines pyroptotic cell death. Nature 526 (7575), 660–665. 10.1038/nature15514 26375003

[B133] Song Z. S. Shao H. Chen Y. Q. Zhang R. (2018). Expression and significance of NLRP3/IL-1β/TGF-β(1) signal axis in rat model of silicosis pulmonary fibrosis. Zhonghua Lao Dong Wei Sheng Zhi Ye Bing Za Zhi 36 (11), 819–823. 10.3760/cma.j.issn.1001-9391.2018.11.005 30646643

[B134] Song Z. S. Zhang R. Zhang J. Shao H. (2020). Inhibition of NLRP3 inflammasome activation on the inflammatory response of macrophage induced by silica dust. Zhonghua Lao Dong Wei Sheng Zhi Ye Bing Za Zhi 38 (6), 406–409. 10.3760/cma.j.cn121094-20190927-00456 32629566

[B135] Sordi M. B. Magini R. S. Panahipour L. Gruber R. (2021). Pyroptosis-mediated periodontal disease. Int. J. Mol. Sci. 23 (1), 372. 10.3390/ijms23010372 35008798 PMC8745163

[B136] Swanson K. V. Deng M. Ting J. P. (2019). The NLRP3 inflammasome: Molecular activation and regulation to therapeutics. Nat. Rev. Immunol. 19 (8), 477–489. 10.1038/s41577-019-0165-0 31036962 PMC7807242

[B137] Taabazuing C. Y. Okondo M. C. Bachovchin D. A. (2017). Pyroptosis and apoptosis pathways engage in bidirectional crosstalk in monocytes and macrophages. Cell Chem. Biol. 24 (4), 507–514. 10.1016/j.chembiol.2017.03.009 28392147 PMC5467448

[B138] Tan S. Chen S. (2021a). Macrophage autophagy and silicosis: Current perspective and latest insights. Int. J. Mol. Sci. 22 (1), 453. 10.3390/ijms22010453 33466366 PMC7795780

[B139] Tan S. Chen S. (2021b). The mechanism and effect of autophagy, apoptosis, and pyroptosis on the progression of silicosis. Int. J. Mol. Sci. 22 (15), 8110. 10.3390/ijms22158110 34360876 PMC8348676

[B140] Tanaka S. Mizushina Y. Kato Y. Tamura M. Shiroishi T. (2013). Functional conservation of Gsdma cluster genes specifically duplicated in the mouse genome. G3 (Bethesda) 3 (10), 1843–1850. 10.1534/g3.113.007393 23979942 PMC3789809

[B141] Tham Y. K. Bernardo B. C. Ooi J. Y. Weeks K. L. McMullen J. R. (2015). Pathophysiology of cardiac hypertrophy and heart failure: Signaling pathways and novel therapeutic targets. Arch. Toxicol. 89 (9), 1401–1438. 10.1007/s00204-015-1477-x 25708889

[B142] Thornberry N. A. Rano T. A. Peterson E. P. Rasper D. M. Timkey T. Garcia-Calvo M. (1997). A combinatorial approach defines specificities of members of the caspase family and granzyme B. Functional relationships established for key mediators of apoptosis. J. Biol. Chem. 272 (29), 17907–17911. 10.1074/jbc.272.29.17907 9218414

[B143] Tian J. Wang B. Xie B. Liu X. Zhou D. Hou X. (2022). Pyroptosis inhibition alleviates potassium oxonate- and monosodium urate-induced gouty arthritis in mice. Mod. Rheumatol. 32 (1), 221–230. 10.1080/14397595.2021.1899569 33705241

[B144] Tsuchiya K. Nakajima S. Hosojima S. Thi Nguyen D. Hattori T. Manh Le T. (2019). Caspase-1 initiates apoptosis in the absence of gasdermin D. Nat. Commun. 10 (1), 2091. 10.1038/s41467-019-09753-2 31064994 PMC6505044

[B145] Wandel M. P. Kim B. H. Park E. S. Boyle K. B. Nayak K. Lagrange B. (2020). Guanylate-binding proteins convert cytosolic bacteria into caspase-4 signaling platforms. Nat. Immunol. 21 (8), 880–891. 10.1038/s41590-020-0697-2 32541830 PMC7381384

[B146] Wang J. C. Shi Q. Zhou Q. Zhang L. L. Qiu Y. P. Lou D. Y. (2022a). Sapidolide A alleviates acetaminophen-induced acute liver injury by inhibiting NLRP3 inflammasome activation in macrophages. Acta Pharmacol. Sin. 43 (8), 2016–2025. 10.1038/s41401-021-00842-x 35022542 PMC9343373

[B147] Wang J. C. Yan H. Chen X. Lee J. Sun J. Liu G. (2022b). Caspase-8 is involved in pyroptosis, necroptosis and the maturation and release of IL-1β in Aspergillus fumigatus keratitis. Int. Immunopharmacol. 113, 109275. 10.1016/j.intimp.2022.109275 36274488

[B148] Wang J. Jiang S. Zhang Y. Li P. Wang K. (2019b). The multifaceted roles of pyroptotic cell death pathways in cancer. Cancers (Basel) 11 (9), 1313. 10.3390/cancers11091313 31492049 PMC6770479

[B149] Wang J. Wu Q. Yu J. Cao X. Xu Z. (2019a). miR-125a-5p inhibits the expression of NLRP3 by targeting CCL4 in human vascular smooth muscle cells treated with ox-LDL. Exp. Ther. Med. 18 (3), 1645–1652. 10.3892/etm.2019.7717 31410121 PMC6676174

[B150] Wang Y. Gao W. Shi X. Ding J. Liu W. He H. (2017). Chemotherapy drugs induce pyroptosis through caspase-3 cleavage of a gasdermin. Nature 547 (7661), 99–103. 10.1038/nature22393 28459430

[B151] Wang Y. Song X. Li Z. Liu N. Yan Y. Li T. (2020). MicroRNA-103 protects coronary artery endothelial cells against H(2)O(2)-induced oxidative stress via BNIP3-mediated end-stage autophagy and antipyroptosis pathways. Oxid. Med. Cell Longev. 2020, 8351342. 10.1155/2020/8351342 32190178 PMC7071805

[B152] Wang Y. Zhai S. Wang H. Jia Q. Jiang W. Zhang X. (2013). Absent in melanoma 2 (AIM2) in rat dental pulp mediates the inflammatory response during pulpitis. J. Endod. 39 (11), 1390–1394. 10.1016/j.joen.2013.07.003 24139260

[B153] Watanabe A. Sohail M. A. Gomes D. A. Hashmi A. Nagata J. Sutterwala F. S. (2009). Inflammasome-mediated regulation of hepatic stellate cells. Am. J. Physiol. Gastrointest. Liver Physiol. 296 (6), G1248–G1257. 10.1152/ajpgi.90223.2008 19359429 PMC2697939

[B154] Wei P. Z. Szeto C. C. (2019). Mitochondrial dysfunction in diabetic kidney disease. Clin. Chim. Acta 496, 108–116. 10.1016/j.cca.2019.07.005 31276635

[B155] Williams P. A. Marsh-Armstrong N. Howell G. R. Lasker I. I. o. A. Glaucomatous Neurodegeneration P. (2017). Neuroinflammation in glaucoma: A new opportunity. Exp. Eye Res. 157, 20–27. 10.1016/j.exer.2017.02.014 28242160 PMC5497582

[B156] Wu C. Orozco C. Boyer J. Leglise M. Goodale J. Batalov S. (2009). BioGPS: An extensible and customizable portal for querying and organizing gene annotation resources. Genome Biol. 10 (11), R130. 10.1186/gb-2009-10-11-r130 19919682 PMC3091323

[B157] Wu G. Zhang D. Yang L. Wu Q. Yuan L. (2022). MicroRNA-200c-5p targets NIMA Related Kinase 7 (NEK7) to inhibit NOD-like receptor 3 (NLRP3) inflammasome activation, MODE-K cell pyroptosis, and inflammatory bowel disease in mice. Mol. Immunol. 146, 57–68. 10.1016/j.molimm.2022.03.121 35447415

[B158] Wu J. Lin S. Chen W. Lian G. Wu W. Chen A. (2023). TNF-α contributes to sarcopenia through caspase-8/caspase-3/GSDME-mediated pyroptosis. Cell Death Discov. 9 (1), 76. 10.1038/s41420-023-01365-6 36823174 PMC9950087

[B159] Wu L. M. Wu S. G. Chen F. Wu Q. Wu C. M. Kang C. M. (2020). Atorvastatin inhibits pyroptosis through the lncRNA NEXN-AS1/NEXN pathway in human vascular endothelial cells. Atherosclerosis 293, 26–34. 10.1016/j.atherosclerosis.2019.11.033 31830726

[B160] Wu M. Han W. Song S. Du Y. Liu C. Chen N. (2018). NLRP3 deficiency ameliorates renal inflammation and fibrosis in diabetic mice. Mol. Cell Endocrinol. 478, 115–125. 10.1016/j.mce.2018.08.002 30098377

[B161] Xi H. Zhang Y. Xu Y. Yang W. Y. Jiang X. Sha X. (2016). Caspase-1 inflammasome activation mediates homocysteine-induced pyrop-apoptosis in endothelial cells. Circ. Res. 118 (10), 1525–1539. 10.1161/CIRCRESAHA.116.308501 27006445 PMC4867131

[B162] Xia X. Wang X. Zheng Y. Jiang J. Hu J. (2019). What role does pyroptosis play in microbial infection? J. Cell Physiol. 234 (6), 7885–7892. 10.1002/jcp.27909 30537070

[B163] Xiao J. Wang C. Yao J. C. Alippe Y. Xu C. Kress D. (2018). Gasdermin D mediates the pathogenesis of neonatal-onset multisystem inflammatory disease in mice. PLoS Biol. 16 (11), e3000047. 10.1371/journal.pbio.3000047 30388107 PMC6235378

[B164] Xu B. Jiang M. Chu Y. Wang W. Chen D. Li X. (2018a). Gasdermin D plays a key role as a pyroptosis executor of non-alcoholic steatohepatitis in humans and mice. J. Hepatol. 68 (4), 773–782. 10.1016/j.jhep.2017.11.040 29273476

[B165] Xu B. Zheng L. Hu Y. W. Wang Q. (2018b). Pyroptosis and its relationship to atherosclerosis. Clin. Chim. Acta 476, 28–37. 10.1016/j.cca.2017.11.005 29129476

[B166] Xu W. Che Y. Zhang Q. Huang H. Ding C. Wang Y. (2021). Apaf-1 pyroptosome senses mitochondrial permeability transition. Cell Metab. 33 (2), 424–436.e10. 10.1016/j.cmet.2020.11.018 33308446

[B167] Yan W. Chang Y. Liang X. Cardinal J. S. Huang H. Thorne S. H. (2012). High-mobility group box 1 activates caspase-1 and promotes hepatocellular carcinoma invasiveness and metastases. Hepatology 55 (6), 1863–1875. 10.1002/hep.25572 22234969 PMC4610360

[B168] Yang D. He Y. Munoz-Planillo R. Liu Q. Nunez G. (2015). Caspase-11 requires the pannexin-1 channel and the purinergic P2X7 pore to mediate pyroptosis and endotoxic shock. Immunity 43 (5), 923–932. 10.1016/j.immuni.2015.10.009 26572062 PMC4795157

[B169] Yang F. Wang Z. Wei X. Han H. Meng X. Zhang Y. (2014). NLRP3 deficiency ameliorates neurovascular damage in experimental ischemic stroke. J. Cereb. Blood Flow. Metab. 34 (4), 660–667. 10.1038/jcbfm.2013.242 24424382 PMC3982086

[B170] Yang M. Lv H. Liu Q. Zhang L. Zhang R. Huang X. (2020). Colchicine alleviates cholesterol crystal-induced endothelial cell pyroptosis through activating AMPK/SIRT1 pathway. Oxid. Med. Cell Longev. 2020, 9173530. 10.1155/2020/9173530 32733639 PMC7378601

[B171] Youm Y. H. Nguyen K. Y. Grant R. W. Goldberg E. L. Bodogai M. Kim D. (2015). The ketone metabolite beta-hydroxybutyrate blocks NLRP3 inflammasome-mediated inflammatory disease. Nat. Med. 21 (3), 263–269. 10.1038/nm.3804 25686106 PMC4352123

[B172] Yu J. Kang M. J. Kim B. J. Kwon J. W. Song Y. H. Choi W. A. (2011). Polymorphisms in GSDMA and GSDMB are associated with asthma susceptibility, atopy and BHR. Pediatr. Pulmonol. 46 (7), 701–708. 10.1002/ppul.21424 21337730

[B173] Yu X. Ma X. Lin W. Xu Q. Zhou H. Kuang H. (2021). Long noncoding RNA MIAT regulates primary human retinal pericyte pyroptosis by modulating miR-342-3p targeting of CASP1 in diabetic retinopathy. Exp. Eye Res. 202, 108300. 10.1016/j.exer.2020.108300 33065089

[B174] Zaslona Z. Flis E. Wilk M. M. Carroll R. G. Palsson-McDermott E. M. Hughes M. M. (2020). Caspase-11 promotes allergic airway inflammation. Nat. Commun. 11 (1), 1055. 10.1038/s41467-020-14945-2 32103022 PMC7044193

[B175] Zhang A. Wang P. Ma X. Yin X. Li J. Wang H. (2015). Mechanisms that lead to the regulation of NLRP3 inflammasome expression and activation in human dental pulp fibroblasts. Mol. Immunol. 66 (2), 253–262. 10.1016/j.molimm.2015.03.009 25863775

[B176] Zhang E. Chen X. Gueydan C. Han J. (2018b). Plasma membrane changes during programmed cell deaths. Cell Res. 28 (1), 9–21. 10.1038/cr.2017.133 29076500 PMC5752838

[B177] Zhang E. Yang Y. Chen S. Peng C. Lavin M. F. Yeo A. J. (2018a). Bone marrow mesenchymal stromal cells attenuate silica-induced pulmonary fibrosis potentially by attenuating Wnt/β-catenin signaling in rats. Stem Cell Res. Ther. 9 (1), 311. 10.1186/s13287-018-1045-4 30428918 PMC6234553

[B178] Zhang J. Y. Jiang Y. X. Yang Y. C. Liu J. Y. Huo C. Ji X. L. (2021b). Cigarette smoke extract induces pyroptosis in human bronchial epithelial cells through the ROS/NLRP3/caspase-1 pathway. Life Sci. 269, 119090. 10.1016/j.lfs.2021.119090 33465393

[B179] Zhang J. Y. Shang X. Jin S. Ma Z. Wang H. Ao N. (2021c). Vitamin D ameliorates high-fat-diet-induced hepatic injury via inhibiting pyroptosis and alters gut microbiota in rats. Arch. Biochem. Biophys. 705, 108894. 10.1016/j.abb.2021.108894 33965368

[B180] Zhang J. Y. Zhou B. Sun R. Y. Ai Y. L. Cheng K. Li F. N. (2021a). The metabolite α-KG induces GSDMC-dependent pyroptosis through death receptor 6-activated caspase-8. Cell Res. 31 (9), 980–997. 10.1038/s41422-021-00506-9 34012073 PMC8410789

[B181] Zhang Z. Zhang Y. Xia S. Kong Q. Li S. Liu X. (2020). Gasdermin E suppresses tumour growth by activating anti-tumour immunity. Nature 579 (7799), 415–420. 10.1038/s41586-020-2071-9 32188940 PMC7123794

[B182] Zhao J. Wei K. Jiang P. Chang C. Xu L. Xu L. (2022). Inflammatory response to regulated cell death in gout and its functional implications. Front. Immunol. 13, 888306. 10.3389/fimmu.2022.888306 35464445 PMC9020265

[B183] Zhao Q. Hao C. Wei J. Huang R. Li C. Yao W. (2021). Bone marrow-derived mesenchymal stem cells attenuate silica-induced pulmonary fibrosis by inhibiting apoptosis and pyroptosis but not autophagy in rats. Ecotoxicol. Environ. Saf. 216, 112181. 10.1016/j.ecoenv.2021.112181 33848736

[B184] Zhaolin Z. Jiaojiao C. Peng W. Yami L. Tingting Z. Jun T. (2019). OxLDL induces vascular endothelial cell pyroptosis through miR-125a-5p/TET2 pathway. J. Cell Physiol. 234 (5), 7475–7491. 10.1002/jcp.27509 30370524

[B185] Zheng M. Karki R. Vogel P. Kanneganti T. D. (2020). Caspase-6 is a key regulator of innate immunity, inflammasome activation, and host defense. Cell 181 (3), 674–687. 10.1016/j.cell.2020.03.040 32298652 PMC7425208

[B186] Zhou Z. He H. Wang K. Shi X. Wang Y. Su Y. (2020). Granzyme A from cytotoxic lymphocytes cleaves GSDMB to trigger pyroptosis in target cells. Science 368 (6494), eaaz7548. 10.1126/science.aaz7548 32299851

[B187] Zhu H. X. Gao J. L. Zhao M. M. Li R. Tian Y. X. Wang X. (2016). Effects of bone marrow-derived mesenchymal stem cells on the autophagic activity of alveolar macrophages in a rat model of silicosis. Exp. Ther. Med. 11 (6), 2577–2582. 10.3892/etm.2016.3200 27284351 PMC4887925

[B188] Zhu Y. Zhao H. Lu J. Lin K. Ni J. Wu G. (2021). Caspase-11-Mediated hepatocytic pyroptosis promotes the progression of nonalcoholic steatohepatitis. Cell Mol. Gastroenterol. Hepatol. 12 (2), 653–664. 10.1016/j.jcmgh.2021.04.009 33894425 PMC8261017

[B189] Zychlinsky A. Prevost M. C. Sansonetti P. J. (1992). Shigella flexneri induces apoptosis in infected macrophages. Nature 358 (6382), 167–169. 10.1038/358167a0 1614548

